# Severity-aware hierarchical federated learning for privacy-preserving indoor emergency recognition in BLE-based medical IoT environments

**DOI:** 10.3389/fmedt.2026.1900013

**Published:** 2026-08-25

**Authors:** Chembon Rajeendran Karthik, Shyam Venkatraman, Hemalatha K

**Affiliations:** School of Computer Science and Engineering, Vellore Institute of Technology, Chennai, Tamil Nadu, India

**Keywords:** BLE, emergency detection, ESP32, federated learning, FedProx, iBeacon, indoor localization, IoT

## Abstract

Indoor safety in GPS-denied environments such as hospitals remains critically hindered by the lack of data-local, context-aware emergency localization systems. This paper presents a complete three-layer IoT framework, namely Medical Indoor BLE Emergency System (MIBES), integrating BLE 4.2 and Federated Learning (FL) for real-time emergency detection, localization and severity weighted aggregation of manually triggered emergency alerts. Built upon an ESP32-based hardware architecture, the system employs BLE Safety Tags with long-press emergency triggers and Anchor nodes enhanced with Exponential Moving Average (EMA) smoothing and utilizing the User Datagram Protocol (UDP) for data encapsulation and transport. The key novelty of this work lies in the proposed Transformer-based Severity-Aware Hierarchical Federated Learning (SA-HFL) framework. This system jointly addresses class imbalance and statistical data heterogeneity in emergency localization through the integration of Focal Loss. This design lets important emergency classes get priority without ruining the overall model generalisation. The Transformer model achieves an accuracy of 99.52%, recall of 99.08% and a MCC of 0.9904 when evaluated on a real-world BLE dataset via 5-fold cross-validation and numerically outperforms the standard FedAvg and FedProx model baselines with performance gains that is supported by moderate to large effect sizes though not by statistically significant paired tests. Communication efficiency remains high with only 150,849 parameters exchanged in each aggregation round. Consequently, the results validate the proposed architecture as a scalable, privacy-preserving solution for reliable emergency alert localization and prioritization across healthcare critical infrastructure.

## 1 Introduction

Recent studies in IoT privacy indicate that straightforward, real-time security measures frequently yield the best results. Modern methods focus on systems that are aware of the environment and the user and automatically change privacy settings based on the situation [1]. This is instead of relying on static configurations. At the same time, recent advancements in federated learning have started to change the way collaborative systems can learn from data without having to centralize it. This has brought new attention to healthcare settings where the risks of exposing data are very high [2]. The reason GPS doesn’t work in these places is because of the laws of physics. Building materials, multi-floor layouts, and metal objects all weaken satellite signals so much that it is impossible to use GPS for emergency response indoors [3]. Instead, systems need to use other wireless sensing methods like WiFi, BLE, or UWB that use less energy and don’t need a constant internet connection to work [4].

While these modalities are available, the core challenge remains a fundamental limitation in signal processing. Raw RSSI signals are inherently noisy, suffering from multipath reflections and body shadowing that introduce rapid fluctuations [5]. These fluctuations can mislead decision logic, leading to false assignments or missed emergencies in real-world fingerprinting deployments [6]. This is particularly critical in healthcare tracking applications, where BLE beacons are increasingly used for patient localization [7]. To address the unreliability of raw signals, recent systems have integrated machine learning directly on wearable safety devices [8]. Furthermore, to produce reliable distance estimates, robust filtering techniques, such as Kalman filters or sophisticated AoA and RSSI fusion have become essential [9].

In addition to signal instability, there is a more basic semantic gap in the design of current BLE frameworks. Commercial platforms often lack the transparency and configurability necessary for real-time safety, which requires the development of custom, reconfigurable wearable devices for ambient assisted living systems [10]. This creates systemic gaps in the overall field of Bluetooth indoor localization [11].

What is genuinely needed is a system that bridges these domains is a lightweight, recalibration-free decision mechanism that provides real-time signal intelligence without compromising privacy. Building upon these limitations, this paper proposes Medical Indoor BLE Emergency System (MIBES), a three-tier, context-aware framework that integrates hardware edge intelligence with hierarchical federated learning. The system utilizes an ESP32-based wearable BLE Safety Tag with a hardware-gated emergency trigger, BLE Anchor Nodes for noise-resilient EMA smoothing (α=0.25), and a Gateway Node for temporal aggregation. In MIBES, the two sub-tasks are being differentiated to prevent confusion. Model learning is not used in zone-level location resolution, which is a deterministic, rule-based process carried out at the Gateway Node utilizing strongest-RSSI selection. The proposed SA-HFL classifier uses the resolved zone, anchor identification, RSSI, sequence, user, and timestamp as input characteristics to predict the binary status label; this is the only learning task.

MIBES is designed to reliably detect, localize and to prioritize manually triggered emergency alerts rather than clinically identify the nature of a medical event. This physical infrastructure feeds directly into a Transformer-based Severity-Aware Hierarchical Federated Learning (SA-HFL) model that trains locally across distributed devices.

The main contributions of the proposed work can be summarized as follows:

- Developed a hardware-validated BLE 4.2 emergency localization system on ESP32 namely the MIBES framework. This process uses passive anchor scanning, EMA-smoothed RSSI, UDP Protocol, and a 1 s gateway aggregation, operating completely without GPS or cloud dependency.
- Implemented a dual-layer false alarm suppression, combining a 2 s long-press firmware gate with sequence de-duplication. Also, implemented a 1.5-second cool-down to virtually eliminate accidental emergency triggers.
- Proposed a Severity-Aware Hierarchical Federated Learning framework using a Transformer backbone and Focal Loss (α=0.75,γ=2) to prioritize learning from emergency alert rich clients, improving the reliability of alarm classification under non-IID data conditions.
- Designed a data-local pipeline where only anonymized, smoothed metadata is processed and federated aggregation is restricted to local network devices, reducing raw-data exposure.
- Conducted ablation and reliability study experiments across five federated configurations, showing attention-based aggregation achieved the most stable performance with a recall standard deviation of 0.0042.

The remainder of the paper is structured as follows. Section 2 reviews the related work that have been published in BLE based indoor localization, federated learning in healthcare and emergency detection systems and identifying the major gaps in them. Section 3 describes the proposed methodology in a detailed manner from dataset generation to detailed explanation of the system architecture. Section 4 shows the results that are obtained from the proposed methodology. Section 5 is the conclusion and Section 6 discusses the future improvements that can be done on the proposed system.

## 2 Literature survey

Milano et al. [3] investigated how the Received Signal Strength Indicator (RSSI) from BLE 5.0 could improve indoor position tracking. They reviewed methods such as applying path-loss models (finding typical values around (A≈−52) dBm and (n≈2.2), using Kalman filtering, combining RSSI readings from multiple channels (using the max, mean, or Maximal Ratio Combining—MRC), using numerical optimization like Nelder-Mead, and experimenting with machine learning, either with a FNN helping out or for the entire process. They discovered that the initial location error was roughly 1.6 m in their own experiments, which were conducted in a 30 $m^{2}$ lab with six anchor points (when they didn’t employ any combination or filtering, instances 1-2). Simply combining the RSSI readings (cases 3–6) reduced this mistake to around 1 meter (a gain of about 35%). The two most important aspects that are still lacking are a clear understanding of the optimal mix of different methods and an accurate comparison between them. WiFi, BLE, UWB, and IMU are highlighted by Leitch et al. [4] as essential indoor localization technologies using RSS, CSI, RTT, AoA, fingerprinting, trilateration, and PDR techniques. While WiFi provides scalability and UWB attains centimeter-level accuracy, BLE guarantees low cost and IMU permits tracking without infrastructure.

Indoor localization research employs a variety of technologies, such as satellite, inertial, magnetic, acoustic, optical, and RF (Wi-Fi, BLE, UWB, RFID), according to Obeidat et al. [5]. Additionally, they use a variety of algorithms, including dead reckoning, proximity, fingerprinting, lateration (RSS/TOA/TDOA), and angulation (AOA). Although precision has increased to the meter or even centimeter level when hybrid and machine-learning technologies are used, there are still many obstacles to overcome. These include minimizing multipath interference and Non-Line-of-Sight (NLOS) problems, guaranteeing synchronization, preserving scalability and energy economy, and dynamically updating radio-maps in intricate interior environments. Safwat et al. [6] created an indoor locating system based on deterministic KNN/WKNN algorithms and RSSI using BLE fingerprints. They gathered data on a grid and used averaging for filtering in order to create the offline RSSI fingerprints. They employed inverse weighting and Euclidean distance for online matching. Their results show that WKNN is superior at lowering mean and standard deviation errors, especially when handling interference and multipath problems. Nevertheless, there are still issues with the system’s capacity to grow, adjust to shifting conditions, and take use of more sophisticated machine learning techniques. Shi et al. [11] investigated Bluetooth indoor localization to categorize techniques into triangulation, fingerprinting, and proximity using RSSI, CSI, AoA, and ToF. RSSI-based systems predominate because they are inexpensive but have multipath and instability, while CSI increases accuracy but necessitates protocol modifications. The requirement for reliable, flexible, and low-overhead solutions is highlighted by significant gaps such as environmental sensitivity, latency–accuracy trade-offs, scalability, and high maintenance costs.

A study that mainly used Wi-Fi/BLE fingerprinting with methods including SVM, K-means, fuzzy matching, PSO, and backpropagation neural networks was proposed by Karamat et al. [12]. However, multipath and NLOS effects cause RSSI instability, which limits accuracy. Temporal modeling and noise filtering are not combined in current methods. Kalman filtering, KNN classification, and LSTM-based recurrent networks are used in this work to solve this, and the average error is 61.29 cm, a 56% improvement. Indoor positioning systems that use existing Bluetooth Low Energy (BLE) and Received Signal Strength (RSS) often rely on measuring signal loss to figure out location (lateration). Pascacio et al. [13] had mentioned that the method is unreliable because of things like signals being blocked (Non-Line-of-Sight or NLOS), not enough positioning beacons, erratic signal changes, and differences between hardware. Previous studies have tried to boost accuracy using different techniques such as least-squares lateration, modeling path loss with artificial neural networks (ANNs), or creating cooperative frameworks. But, not much work has combined BLE-based cooperation with neural networks. The main hurdle is accurately figuring out distances when devices are varied and the arrangement of positioning beacons is poor.

Existing BLE-based proximity detection methods rely on RSSI regression, filtering, or deep learning with IMU. The proposed PRID framework by Tianlang et al. [14] mitigated multipath via RSSI histogramization and reduces IMU overfitting through carriage state regularization, achieving robust, memory-efficient proximity classification but inadequately address multipath distortion and biased IMU training data, leading to poor generalization. Current research on indoor positioning using Bluetooth Low Energy (BLE) uses techniques like probabilistic fingerprinting, Monte Carlo filters, trilateration, and neural networks. These methods usually have an average error of about 3.1 to 3.7 meters. However, there are still some missing pieces, especially when it comes to the detailed design of capturing and filtering the Received Signal Strength Indicator (RSSI) data, making the system work even when some data is missing, and testing the system with real-world movement paths. Ferrero et al. [15] tried to fix these issues by using a better, window-based filtering approach and several fine-tuned models, which brought the average error down to 1.9 meters. Still, their work is limited because it depends heavily on a specific dataset and doesn’t easily work in new, different environments. Khor et al. [16] found a way to pinpoint locations indoors. They used the signal strength (RSSI) from Wi-Fi and Bluetooth Low Energy (BLE) as a fingerprint, along with statistical methods like weighted k-nearest neighbors (kNN), Support Vector Machines (SVM), and layered trees, as well as complex networks. They also used geometric methods like trilateration and Ultra-Wideband (UWB) timing methods (ToA/ToF). Even though they got better accuracy, there are still problems: signals bounce around (multipath fading), they are unreliable, the equipment is expensive, it needs a lot of hardware, and many phones aren’t compatible. Consequently, the development of more economical and reliable approaches based on BLE is necessary.

Surian et al. [17] in their study on RNSI localization utilizing BLE, identified a number of difficulties. The tests only included one person and were single-run per setting, which limits the system’s potential for scalability and the ease with which the results may be replicated. The results are less broadly applicable because the study was restricted to just two different situations. The optimal location for the beacons and the potential impact of many users’ interference on performance were not examined. Additionally, the occasional failure to pinpoint a location indicates that it is still difficult to sustain dependable performance in extremely busy or quickly changing surroundings. Ghaemifar et al. [18] in their review of BLE-based Indoor Positioning Systems (IPS), covered geometric (trilateration, multilateration) and fingerprinting techniques using RSSI, ToA, TDoA, AoA, RTT, CSI, and hybrid fusion with Wi-Fi, UWB, IMU, and LiDAR; machine learning, deep learning, reinforcement learning, and Kalman filtering improve accuracy despite multipath and Non-Line-of-Sight (NLoS) problems. However, challenges based on Standardized datasets, stability in dynamic contexts, synchronization faults, and scaled real-world deployment still persist. Cui et al. [19] looked into edge/fog computing and SDN-MEC setups to make VANETs better. While some employed deep learning techniques like ANN, GCN-GRU, and LSTM to anticipate traffic and human movement, others employed Kalman filters and Markov models to estimate locations. To keep things secret, they even combined blockchain technology with federated learning. However, more advanced methods that are fast, maintain privacy, and manage massive volumes of dispersed data are still required to anticipate the next location. To improve accuracy, security, and computing speed, the proposed PLC architecture integrates blockchain, federated learning, and optimization approaches.

According to Diraco et al. [20], Human Action Recognition (HAR), which uses sensing and advanced learning techniques to identify everyday behaviors in households, healthcare facilities, and urban areas, is essential to smart living. Wearable, ambient, and vision sensors are used in current approaches together with different learning techniques. However, there are still issues with comprehending context, obtaining sufficient data, customizing systems for each user, and safeguarding privacy, which limit how extensively and successfully these systems can be applied in the real world. Alharbey et al. [21] proposed a federated learning-powered elderly monitoring system that uses smartphones with IMU sensors to enable real-time activity recognition, location tracking, and altitude detection. The accuracy was increased from 83.9% to 91.7% by using a Bi-LSTM model that was trained locally and then integrated using FedAvg. Existing work lacks integrated multi-sensor fusion with federated aggregation, which highlights the system’s originality and performance improvements while guaranteeing privacy and scalability. Hema Latha et al. [2] explored the use of federated learning to maintain the privacy of medical image segmentation. To address the lack of labels, they used pseudo-labeling and semi-supervised learning. However, they still faced challenges such as handling noisy pseudo-labels, a wide range of data, and simply a lack of labeled cases. In order to address this, the proposed CBDPL-based FL system cleverly selects reliable pseudo-labels. This improves the segmentation’s accuracy, robustness, and general applicability all while maintaining patient privacy.

In addition to centralized and hierarchical federated techniques, another approach investigates fully decentralized and online federated optimization, in which clients interact directly with nearby nodes via time-varying directed networks and there is no central server. In order to safeguard patient data throughout a virtual decentralized hospital network, Jiang et al. [22] proposed DT-DOFL, a decentralized online federated learning framework for smart healthcare service systems that is powered by digital twins. This framework models client communication as a graph and incorporates differential privacy. DeFedHDP, a fully decentralized online federated learning approach for heart disease prediction, was presented by Wei et al. [23]. In this approach, data holders interact directly with neighbors over time-varying directed graphs without the need for a central server. A differential privacy mechanism is applied to the aggregation strategy, and each participant serves as both a trainer and a collaborator of the models of the other participants. In order to safeguard electronic health records while maximizing communication resources, the same research team proposed AccDFL [24], a doubly accelerated decentralized federated learning algorithm for healthcare IoT networks. It is based on a three-layer framework that divides physical hospital data, communication, and edge-encrypted model interaction.

DP-ZOCOA, a differentially private, gradient-free (zeroth-order) distributed optimization algorithm for constrained problems over time-varying unbalanced directed graphs, was further proposed by Wei et al. [25]. It was expanded into ZOCOA-FL, a decentralized zeroth-order constrained federated learning algorithm, which was validated on both distributed least squares and decentralized federated learning tasks. The online setting was directly addressed in related work by Zhao et al. [26], who proposed a differentially private distributed push-sum one-point bandit dual averaging algorithm for agents that communicate over strongly connected time-varying directed graphs but lack gradient information. This algorithm estimates gradients from single function evaluations while maintaining agent privacy.

### 2.1 Literature gap & contributions

Although there has been research on using Bluetooth Low Energy (BLE) to locate objects indoors, the majority of existing systems mainly focus on improving location accuracy. They accomplish this by using complex AI models (deep learning), measuring distances (trilateration), using “fingerprints” of the location, or cleaning up the signal data (RSSI filtering). These techniques are typically useful for following items, navigating around, or just determining whether something is close. They are not designed to properly identify or localize a manually triggered emergency alert signal under real world constraints. Many of these systems need an excessive amount of processing power, combine data from multiple sensors, or require a lengthy setup process using the fingerprinting method. Because of this, they are not suitable for small, simple wearable devices and are difficult to scale up. In addition, the majority of the BLE systems that are in use in the healthcare industry only track and monitor patients. They lack time-sensitive location zone verification, which is essential for a prompt reaction, as well as built-in features to reliably overlook false alarms and specifically identify crises at the core device level. Additionally, no one has really explored the use of federated learning for BLE indoor emergency detection, despite the fact that it is an effective way to protect privacy when sharing data in healthcare and smart-device systems, particularly when the data from the various location beacons (anchors) is patchy and unevenly distributed. The current federated systems usually just use simple averaging, which fails to take into account the significant risks associated with missing an emergency. In addition to all of this, a fully functional system is being lacked, that has been tested on actual hardware. Wearable emergency buttons, smart signal analysis from the beacons, time-based data aggregation at the network gateway, and a severity-aware, decentralized learning technique would all need to be integrated into a single, unified, privacy-protecting framework.

The Medical Indoor BLE Emergency System (MIBES) was designed in order to address these issues. The three components of this system include a firmware-controlled mechanism to initiate emergencies, an EMA-based technique to stabilize signals at the anchor points, a step to group data over time at the gateway, and a smart framework called Severity-Aware Hierarchical Federated Learning (SA-HFL) based on a Transformer model, the system has been thoroughly tested using actual hardware. The proposed approach, in contrast to others, ensures that client updates regarding emergencies are given more weight when combining data, works even when anchor devices are dispersed unevenly (non-IID), and safeguards privacy by providing only the model’s rules rather than the actual patient data. This entire design provides a useful and medically suitable method of identifying indoor emergencies that can expand with the needs by integrating BLE location tracking, emergency understanding, and decentralized intelligence. Table 1 clearly connects the five main issues in existing literature to the precise solutions that the new approach proposes, demonstrating how the technical steps are better to what is now available.

**Table 1:** Comparison of identified literature gaps vs. proposed methodology.

References	Research domain	Identified literature gap	Proposed methodology
Milano et al. [3], Leitch et al. [4], Obeidat et al. [5], Shi et al. [11], Ferrero et al. [15], Ghaemifar et al. [18]	Optimization Focus (Navigation vs. Safety)	Current research is too focused on minimizing exact coordinate errors (usually 1.0–3.7m), which isn’t needed for simple emergency recognition and often fails.	Proposed structure is specially tuned for crucial safety; it uses a 1,000 ms temporal window to aggregate and select the strongest RSSI, making sure zone-level classification is reliable.
Safwat et al. [6], Fabris et al. [9], Beigi et al. [12], He et al. [14]	Computational Overhead & Scalability	Systems rely on intensive signal processing (like Kalman filtering, AoA, or LSTM) or offline grid fingerprinting that needs constant re-calibration, making them unsuitable for ESP32 wearables.	Complex matrix operations and physical re-calibration are avoided by using a simple Exponential Moving Average (EMA, α=0.25) directly on the hardware edge.
Skýpalová et al. [7], Baejah et al. [8], Díaz-Jiménez et al. [10], Surian et al. [17]	False alarm vulnerability in healthcare	Healthcare BLE systems typically only offer passive tracking and don’t have built-in firmware rules to explicitly block environmental noise or accidental triggers.	A physical dual-layer protection mechanism (2,000 ms firmware long-press delay + sequence de-duplication) is introduced to filter noise before it reaches the network.
Hema Latha et al. [2], Alharbey et al. [21], Hema Latha et al. [2]	Imbalanced Medical Risk in Federated Learning	Standard federated models use simple FedAvg weighting ($n_{k}/N$), ignoring the uneven risk of missing emergencies under real-world non-IID conditions.	A Severity-Aware Hierarchical framework (SA-HFL) with Focal Loss (β=0.7) prioritizes updates from emergency-rich anchor devices.
Shaheen et al. [1], Hema Latha et al. [2], Ali et al. [19], Hema Latha et al. [2]	Centralized Data & Privacy Vulnerabilities	Traditional IoT localization systems store raw user data centrally, causing privacy and compliance issues in medical environments.	A data-local learning process shares only model parameters (150,849 weights/round) rather than raw data, reducing but not formally preventing inference risk.

## 3 Methodology

**Figure 1:**
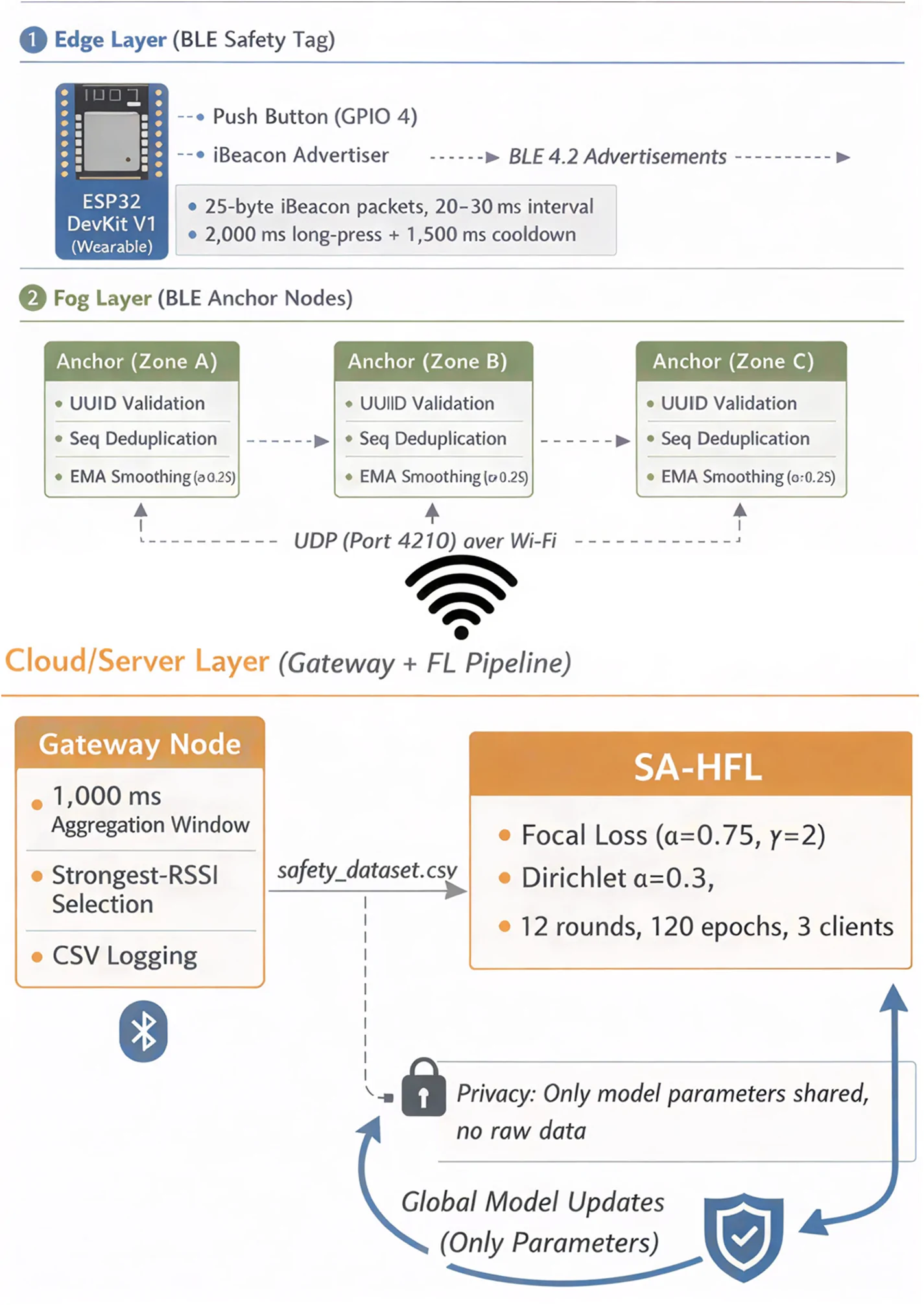
Proposed architecture diagram. Shows the three-tier MIBES system, detailing how information moves from the Edge (BLE Safety Tag), through the Fog (BLE Anchor Nodes smoothing data and sending it via UDP), to the Cloud/Server where the Gateway Node and SA-HFL pipeline are located. The picture highlights that only model parameters are shared during combination, keeping raw patient data local to each device; this data-local design reduces, but does not by itself formally guarantee, privacy against parameter-based inference attacks.

**Figure 2:**
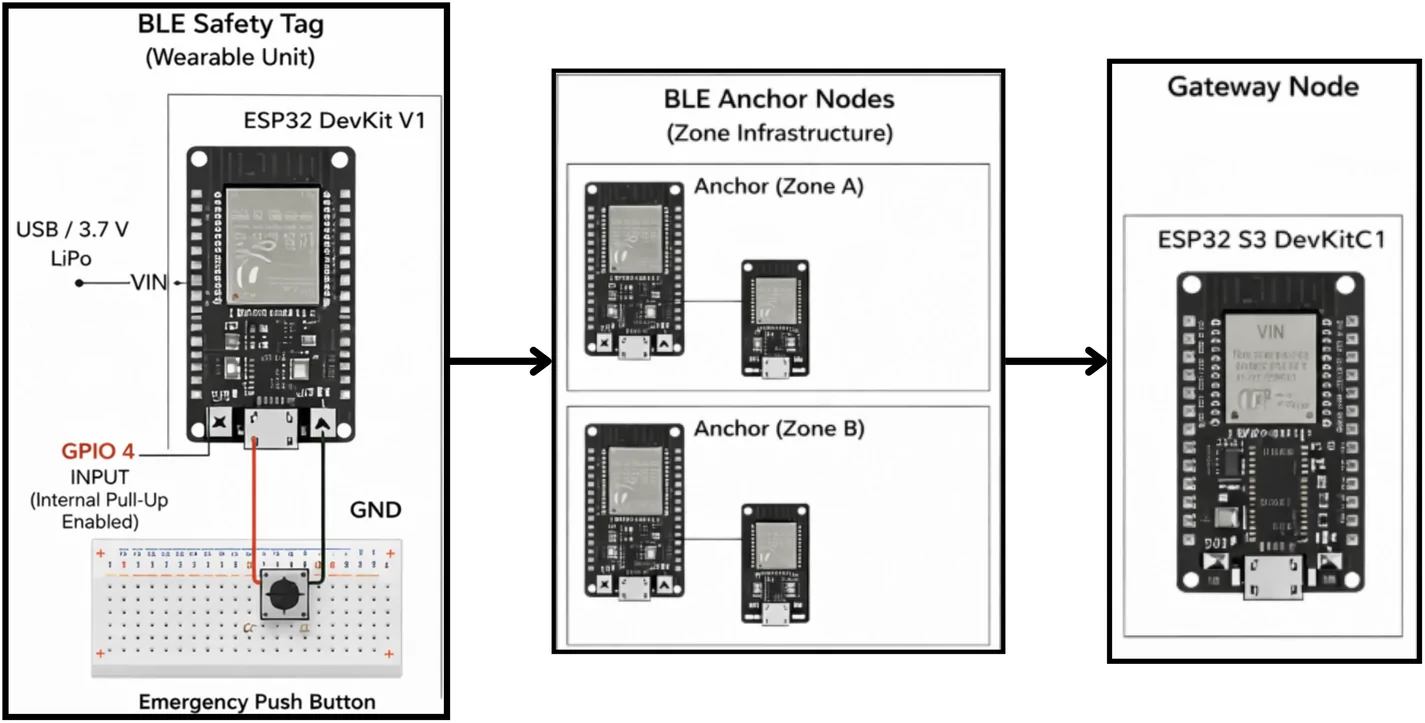
Pin diagram of the proposed architecture. The proposed architecture’s pin diagram The ESP32 Safety Tag’s physical wiring is simple. Using the inbuilt pull-up resistor of the chip, the emergency button connects to GPIO 4. Either a 3.7V LiPo battery or a USB connection provides power.

The proposed system is made up of three main parts as shown in Figure 1. First, when an individual deliberately holds down the button on a wearable BLE Safety Tag near the edge, an emergency iBeacon signal is sent out. Second, three fixed BLE Anchor Nodes in the fog layer detect these signals, verify their validity, eliminate duplicates, and smooth them out. A clean report is then sent to the Gateway Node over UDP. Third, the gateway collects all of the reports in less than a second, determines the emergency location by identifying the anchor with the strongest signal (RSSI), and records the data as a CSV file. The emergency event is itself user initiated when the user presses the safety tag push button, the systems role is to transmit, localize and to classify the alerts rather than to detect the emergency from physiological or environmental sensing. These data are directly integrated into a Federated Learning system that keeps raw data local to the device. The system does not transmit raw sensor data off-device; only model parameters are exchanged during aggregation, so no user-identifying information or raw sensor readings need to leave the local network. It was noted that the parameter sharing alone does not constitute a formal privacy guarantee. Figure 2 shows the pin diagram of a wearable ESP32 device with an emergency push button attached to GPIO 4. When pressed, it sends a signal to surrounding BLE anchor nodes in various zones. These nodes then forward the data to a central ESP32-S3 gateway for processing and observation.

### 3.1 Datasets and workflow

#### 3.1.1 Dataset generation and description

The data used in this study came directly from the actual hardware system’s operation rather than from simulations or made-up data. The suggested method makes use of real, in-field BLE records as opposed to systems relying on theoretical models. This guarantees that the system can manage real-world indoor problems like signal instability and environmental volatility. The 4,000 labeled samples in the collection each represent a particular emergency or common occurrence that the gateway handled. According to the class breakdown, the emergency rate is approximately 0.40, which means that 2,259 samples are normal (status = 0) and 1,741 samples have a positive emergency tag (status = 1). This small class disparity was purposefully maintained to represent genuine inside situations, when real crises occur less frequently than typical occurrences. Instead of just oversampling, Focal Loss was used to address this during the modeling phase. Each data record includes seven fields: timestamp, user, zone, anchor, RSSI, seq, and status, as indicated in Table 2. The structured UDP packet that the anchor nodes deliver to the gateway during live operation is closely correlated with these variables. As categories that represent the actual rooms and hardware pieces in the testbed, the zone and anchor attributes provide spatial context. After determining the strongest RSSI zone, the gateway writes the EMA-smoothed signal strength value to the CSV, which is stored in the RSSI field. The sequence number from the BLE packet is stored in the seq field, which aids in locating and eliminating duplicates in the logged data. The proven truth label, which is derived from the safety flag included in the iBeacon manufacturer-specific data on the tag itself, is provided via the binary status field. So the classification task that is addressed by SA-HFL is the reliable identification and localization of manually triggered emergency alarm signal under realistic BLE noise and non-IID conditions rather than clinical detection of the underlying medical event.

**Table 2:** Dataset fields summary.

Field	Type	Description
Timestamp	Numerical	Unix Timestamp of gateway event
User	Numerical	Pre-assigned user identifier
Zone	Categorical	Named physical zone (e.g., Zone A, Zone B)
Anchor	Categorical	Anchor node identifier
RSSI	Numerical	EMA-smoothed signal strength (dBm)
Seq	Numerical	BLE packet sequence number (missing filled with column mean)
Status	Binary label	0 = Normal, 1 = Emergency

#### 3.1.2 Dataset preprocessing

Binary emergency classification (status = 0/1) is the supervised task that all five federation configurations handle. Here, the zone and anchor data are solely employed as categorical input context rather than as prediction targets. They characterize the result of the gateway’s deterministic RSSI-based zone resolution step. Before putting the 4,000 records into any model, there were always two steps that were done the same way for all the proposed works. OneHotEncoder setup was used to change the categorical features, like zone and anchor. sparse_output was set to False and handle_unknown to “ignore.” This made it easy to see if each unique identifier was yes or no. A StandardScaler was used to process the numerical features, which were Timestamp, User, RSSI, and Sequence. This made sure that the inputs had a mean of zero and a variance of one. This way, no one feature could change the results just because it was big. The final matrix of features had 10 inputs for each sample after the encoding was done and everything was put together. Certain fields like timestamp, user and seq can carry a risk of data leakage, since seq increments specifically when an emergency is triggered at the hardware level and missing values in this field are imputed using column mean. This can lead to a systematic numerical difference between the normal and the emergency classes that is independent of the RSSI based signal intelligence the model is supposed to learn. To evaluate the extent of this risk an additional leakage controlled ablation was conducted in which the timestamp, user and seq fields were removed and only RSSI, zone and anchor fields were retained as inputs. Table 3 shows the whole dataset in detail.

**Table 3:** Class distribution of the full dataset.

Category	Count	Percentage
Total samples	4,000	100%
Normal (status = 0)	2,259	56.475%
Emergency (status = 1)	1,741	43.525%

### 3.2 System hardware architecture

**Figure 3:**
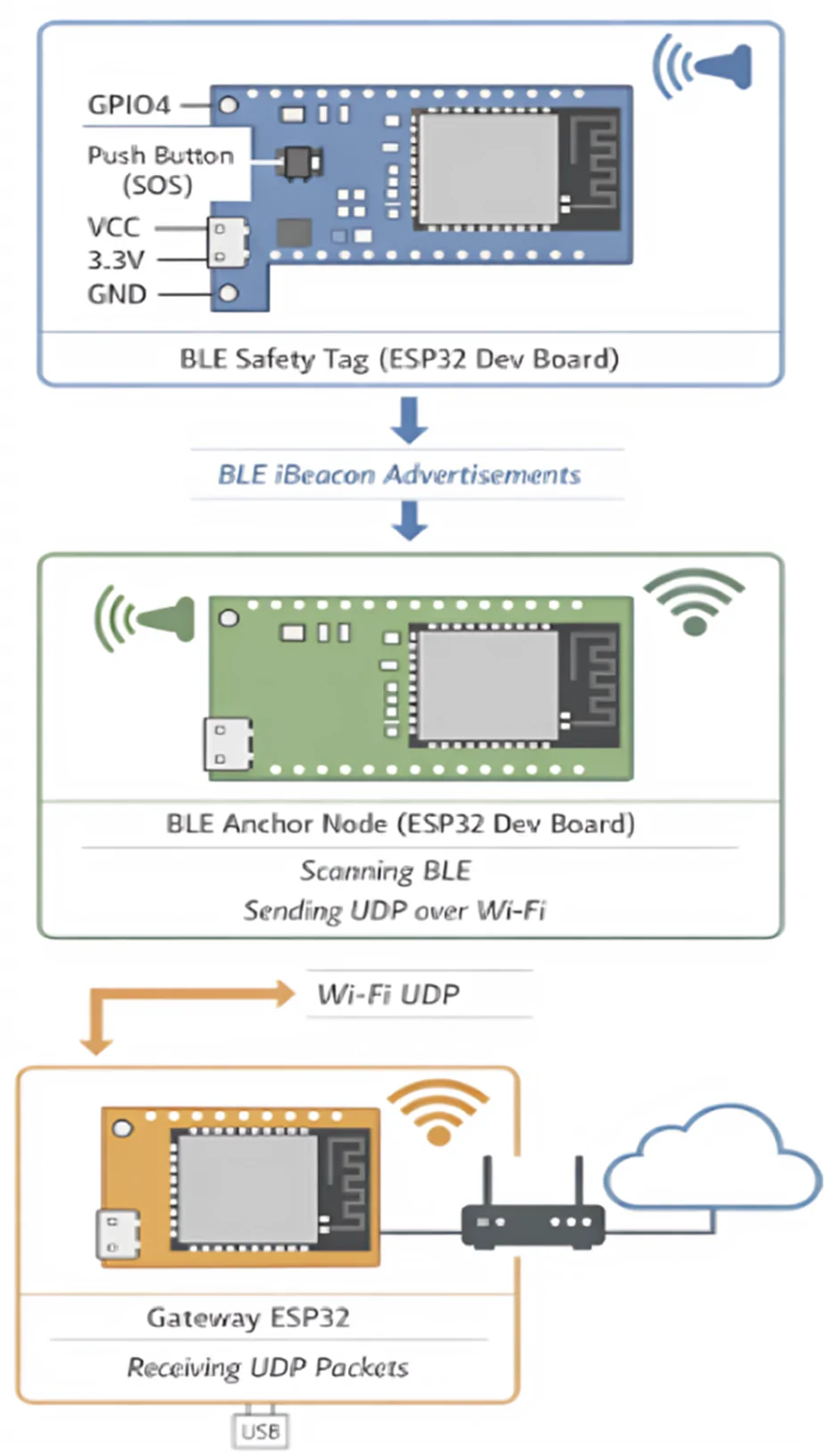
BLE hardware emergency architecture. The BLE Safety Tag sends out iBeacon signals that the ESP32-based BLE Anchor Nodes, which are always listening, pick up. This is the whole process that lets the hardware talk to each other. The central Gateway ESP32-S3 then gets this data over Wi-Fi as UDP packets that are organized. The picture shows how clear the roles are at each step. This makes it easy to add more nodes to the system without having to change the core programming of each one.

The Arduino framework and the PlatformIO build system are used to program all of the ESP32 family microcontrollers that were used to build the system. It doesn’t need GPS or the cloud to work. Figure 3 shows the three-tier architecture that includes a wearable BLE Safety Tag, fixed BLE Anchor Nodes, and a central Gateway Node. This node collects time-windowed reports from all available anchors, compares signal strengths to find the most likely zone of an emergency, and makes structured event records that can be used for both long-term data logging and real-time warnings. Because the roles are so clearly defined, each node only has to do one thing, which makes it easy for the system to grow by adding more anchor nodes. This means that the gateway and tag programming don’t have to be changed. Also, the entire data path, from the first button press to the last logged record, uses anonymous identifiers and smoothed signal data instead of raw information that is sensitive to people or locations. This automatically follows rules for data minimization that are important for hospitals.

#### 3.2.1 BLE safety tag (wearable unit)

The patient wears the Safety Tag like a device that helps in an emergency. This device has an ESP32 DevKit V1 module integrated within it. The Safety Tag uses something called BLE 4.2 to send out signals. It uses the NimBLE Arduino library to do this. It does not initiate connections to a network or look for other devices. This makes the Safety Tag a good choice for things that people wear to stay safe because it does not use a lot of power. The Safety Tag is really good at saving power, which is important, for the Safety Tag. When it starts up, the tag gets NimBLE ready, sets itself up to advertise, and begins sending out 25-byte iBeacon-formatted BLE packets. Every packet carries four bits of information inside the manufacturer-specific data payload: a fixed proximity UUID (for anchors to check), the assigned USER ID, a binary safety status flag (0 = okay, 1 = danger), and an 8-bit sequence number that goes up with each new emergency. Normally, the tag advertises with status = 0 every 20–30 ms (BLE advertising interval values of 32–48 units of 0.625 ms), announcing its presence without kicking off any downstream actions.

To set off the emergency, the patient has to intentionally hold down a push button connected to GPIO 4 for a full 2,000 ms. The ESP32’s built-in pull-up resistor is turned on in the software. The system only registers a real emergency if the button has been held down non-stop for at least 2,000 ms, and if at least 1,500 ms has passed since the last emergency was triggered. This two-part software check is the first way the system stops false alarms. The broadcast data is immediately updated, the sequence number increases, and the safety status is set to 1 when both requirements are satisfied. The fresh emergency packet is then instantly sent out by the tag at the same speed. This ensures that even in a noisy indoor radio environment, neighboring receivers that might be momentarily preoccupied with another packet have a very excellent chance of picking up the signal. The hardware connections on the Safety Tag are intentionally kept to a minimum. A 3.7 V LiPo battery or a USB connection can supply power via the board’s regulator. The sole additional component is a push button that is linked between GPIO 4 and ground (GND) and is typically open. The pull-up resistor ensures that the pin reads low when pressed and high while at rest. There is no need for additional screens, sensors, or radio components. Because of its straightforward design, the gadget is compact, the parts list is inexpensive, and the setup is simple to replicate wherever it is used. All of the timing and identification characteristics of the Safety Tag’s software are initially controlled by a set of predefined values that were specified during program construction. The key firmware parameters utilized in the tag are displayed in Table 4.

**Table 4:** Safety tag firmware constants.

Parameter	Value	Purpose
USER_ID	101	Embedded user identifier
MEASURED_POWER	−59 dBm	TX power @ 1 m (iBeacon)
BUTTON_PIN	GPIO 4	Emergency push button
LONG_PRESS_TIME	2,000 ms	Trigger threshold
MIN_EVENT_GAP_MS	1,500 ms	Cooldown interval
Adv. interval (min)	20 ms	32×0.625 ms
Adv. interval (max)	30 ms	48×0.625 ms
BLE library	NimBLE v1.3.10	Lightweight BLE stack

The tag sends out a 25-byte payload specific to the manufacturer, using the Apple iBeacon format. The standard iBeacon Major, Minor, and Reserved fields are used instead to carry personalized safety information. The device name field is blank so the BLE stack doesn’t use up advertisement space with a name, leaving the entire payload available for manufacturer data, as shown in Table 5.

**Table 5:** iBeacon payload structure.

Byte offset	Field	Size	Value/source
0	iBeacon type	1 byte	0 × 02 (fixed identifier)
1	iBeacon length	1 byte	0 × 15 (21 bytes follow)
2–17	Proximity UUID	16 bytes	System-wide UUID (shared with anchors)
18–19	Major → User ID	2 bytes	Big-endian encoded USER_ID
20–21	Minor → Safety status	2 bytes	0 = Normal, 1 = Emergency
22–23	Reserved → Sequence number	2 bytes	Increments per new emergency event
24	Measured Power	1 byte	−59 dBm (signed int8)

**Figure 4:**
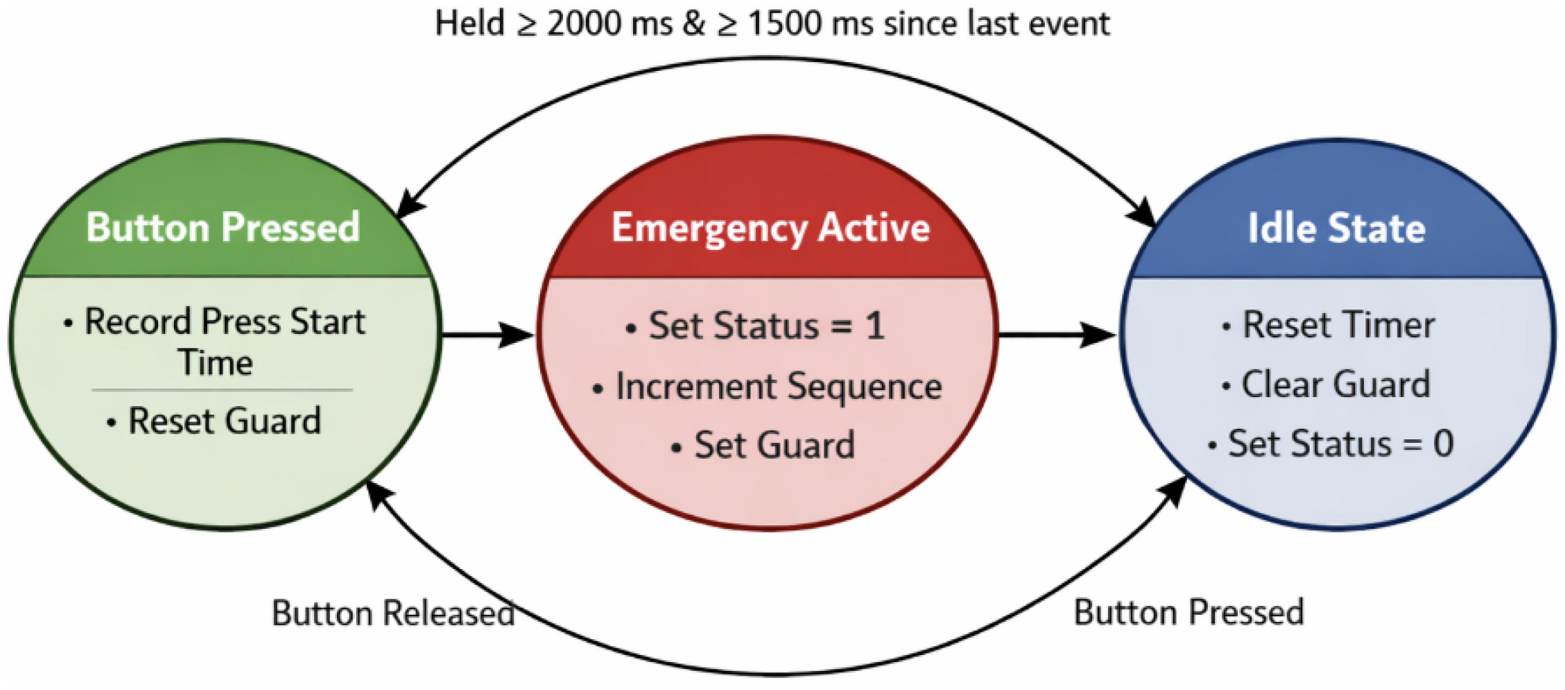
State machine diagram of the states. This describes the full hardware communication path: it begins with the small, wearable BLE Safety Tag sending out iBeacon signals, which are picked up by the ESP32-based BLE Anchor Nodes that are always listening. This data then moves to the main Gateway ESP32-S3, which organizes it into UDP packets via Wi-Fi.

The tag keeps track of four pieces of information: a counter for the sequence, a temporary emergency safeguard flag, a record of when the press started, and a record of the last time an event happened. These variables work together to create a three-state machine, as depicted in Figure 4.

Table 6 shows the hardware connections of the safety tag. Because the system is self-contained, external parts like resistors, sensors, displays, or other modules are not required.

**Table 6:** Safety tag wiring.

Connection	From	To	Notes
Push button	GPIO 4	GND	Internal pull-up enabled; HIGH at rest, LOW when pressed
Power	USB/3.7 V LiPo	ESP32 VIN	Via onboard regulator

#### 3.2.2 BLE anchor nodes (zone infrastructure)

Fixed in predetermined locations around the indoor testbed, three ESP32 DevKit V1 boards serve as BLE Anchor Nodes, each designating a distinct physical area (such as Zone A, Zone B, and so forth). NimBLE is run in passive scanning mode by each anchor node. This indicates that it never sends out scan requests; instead, it simply listens for BLE ads. This method saves radio time for the anchor and avoids interference with the tag’s broadcast. The anchor has a scanning duty cycle of roughly 99%, which is almost constant, when the scan window is set to 99 ms and the scan interval of 100 ms. The firmware of the anchor does a three-step packet filter as soon as a BLE advertisement appears. It starts by searching the advertisement type bytes for the iBeacon structure marker. Second, it rejects any odd BLE devices in the same radio frequency area by verifying that the proximity UUID in the manufacturer-specific payload matches the anticipated, system-wide UUID. Third, it extracts from the unique payload the user_id, safety_status, and seq fields. All packets with normal status are simply discarded; only packets with a safety_status of 1 are handled further.

Since the Safety Tag broadcasts the same emergency packet repeatedly, the anchor uses a deduplication table based on a per-user sequence number to avoid duplicate reporting of a single emergency. If the sequence number (seq value) of an inbound packet already exists for that particular user_id, it is discarded. Only when a packet arrives with a new, higher sequence number is a new emergency report processed.

Each of the three ESP32 DevKit V1 boards covers a distinct physical space and is installed in permanent, pre-surveyed indoor locations. Before delivering structured reports to the gateway, each anchor silently listens for emergency signals, checks them, eliminates duplicates, and smoothes them out. To indicate its physical location, the anchor node has a unique ANCHOR_ID and a corresponding ZONE_NAME. It uses UDP port 4,210 to transmit verified emergency reports to a designated gateway IP. With a 99% passive listening duty cycle and a 100 BLE unit interval (62.5 ms) and 99 BLE unit window (61.875 ms), the scanning arrangement allows for almost constant monitoring. A common 16-byte UUID confirms that the packets are legitimate and validates the system overall, and duplicate filtering is disabled so that repeated advertising can be received for accurate RSSI values.

Before doing anything else with a BLE advertisement, the anchor filters it via a series of processes. It begins by determining if the incoming packet contains manufacturer-specific information. If not, the packet is immediately discarded since it cannot be an iBeacon advertisement. The next step is to confirm that the payload is precisely 25 bytes long, which is the size required for the unique iBeacon format used by the system. Any different-length packet is rejected. The anchor then looks at the first two bytes of the payload, confirming they match the standard iBeacon type ID (0 × 02) and length marker (0 × 15); if they don’t match, it means the packet is from a non-iBeacon BLE device, and it’s dropped. After that, the 16-byte proximity UUID tucked into the payload is checked byte-by-byte against the UUID that the whole system uses. This ensures that only advertisements from the system’s own Safety Tags are accepted, while foreign BLE devices in the same radio space are kept out.

Once the structure checks out, the anchor pulls four pieces of information from the data: the user’s ID from bytes 18 to 19, the safety status light from bytes 20 to 21, the sequence number from bytes 22 to 23, and the raw RSSI number straight from the ESP32’s BLE radio hardware. If that status light isn’t set to 1, the signal is just a regular, non-emergency announcement; it gets quietly thrown out without causing any network activity. If the sequence number matches the last one processed for that user, the signal is recognized as a repeated alert for an emergency already reported, and it’s tossed out for the same reason.

The anchor uses an exponential moving average to smooth the raw RSSI result for packets that pass all previous checks. The utilized Equation 1 is:

$${\text{RSSI}}_{\text{avg}}=(1-\alpha )\;{\text{RSSI}}_{\text{\textbackslash{},prev}}+\alpha \;{\text{RSSI}}_{\text{raw}},\;\alpha =0.25$$ (1)

In this stage, a trustworthy, less noisy signal strength estimate is generated. After that, the anchor creates a seven-data-point structured UDP data package. This includes the zone name, the user’s identity, the emergency status, and the anchor’s identification. Furthermore, the gateway node receives the smoothed RSSI value, a sequence number, and a timestamp via the local WiFi network.

The rapid signal shifts caused by multipath fading, body shadowing, and interior reflections are mitigated by the Exponential Moving Average (EMA) filter. This is achieved through Equation 2:

$${\text{avg}}^{\left(t\right)}=(1-\alpha )\;{\text{avg}}^{\left(t-1\right)}+\alpha \;{\text{RSSI}}^{\left(t\right)}$$ (2)

In firmware-implemented integer arithmetic form:

$$\text{avg}=\frac{\text{avg}\times 3+\text{RSSI}}{4}$$ (3)

where:

- **avg** = maintaining a smoothed RSSI average for each user on the anchor
- **RSSI** = The ESP32 BLE radio’s raw RSSI reading (dBm) for the current packet
- α=0.25(1/4) = smoothing factor; 25% is contributed by each new reading.
- (1−α)=0.75(3/4) = 75% of history is preserved due to the retention factor.

Equation 3 treats each new raw measurement as only one-quarter of the running average, giving it less weight. The large, abrupt fluctuations common to indoor BLE signals, such as multipath fading, individuals obstructing the signal, and reflections off walls, are gradually smoothed down. The raw RSSI is just used to start the average if there isn’t one already (i.e., this is the first packet received). Instead of the raw reading, the final smoothed RSSI is transmitted.

Each UDP data packet an anchor node sends to the gateway contains seven important pieces of information that together describe a single emergency detection. In order for the gateway to determine precisely which device detected the signal, the anchor field contains the name assigned to the reporting anchor at the time the system was constructed, such as ANCHOR_B. The physical region associated with that anchor is named in the zone field, for example, Zone_B. The user field contains the user’s ID, which is taken from the received Bluetooth advertisement’s iBeacon Major field. Because the anchor is intended to transmit only emergency signals and disregard all other signals, the status is always 1. The proximity reading is more steady and dependable because the RSSI field provides the signal intensity after it has been smoothed using an Exponential Moving Average (EMA) rather than the raw, instantaneous data. In order to assist the gateway in grouping reports pertaining to the same emergency, the seq field retains the original serial number from the Bluetooth data. Lastly, the ts field logs the time on the anchor when the data was transmitted, expressed in milliseconds.

UDP was used in instead of TCP. In an emergency, it is more crucial to convey the data fast (low latency) than to make sure every packet arrives (guaranteed delivery). Furthermore, the numerous anchor system inherently provides a backup.

#### 3.2.3 Anchor node networking and data transmission

The anchor sends a structured report to the Gateway over UDP on port 4,210 as soon as an emergency packet passes through all three filters and the RSSI is smoothed. It is a deliberate design decision to use UDP rather than TCP because in an emergency, individuals require speed far more than assured delivery. Additionally, even if one UDP packet is lost, another anchor’s report will reach the gateway inside the same 1,000 ms collection window because to the system’s multianchor backup. AnchorID, Zone Name, Userid, SmoothedRSSI, Seq, and a Unix Timestamp generated on the anchor the moment it transmits are all included in each UDP packet, which is a straightforward, plain text string separated by commas. When it first boots up, the anchor connects to the local WiFi network and maintains that connection. The gateway’s IP address is permanently written into the anchor’s software, letting it send reports without needing any DNS lookup or service finding hassle.

#### 3.2.4 Gateway node (system brain)

The Gateway, which is based on an ESP32 S3 DevKitC 1 module, is the most potent computational node in the system. It sets up a UDP socket on port 4,210 and connects to the same local Wi-Fi network as the anchors, continuously monitoring incoming anchor reports. Grouping incoming UDP messages according to their sequence field is the gateway’s primary job. The gateway starts an aggregation window of 1,000 ms when the first packet for a new sequence number arrives. It gathers all of the reports from all of the anchors who reported the same emergency incident throughout this period. After the window closes, the gateway selects the anchor with the highest (least negative) smoothed RSSI value by looking at all of the reports that were gathered for that sequence. The idea is that the nearest anchor will generate the strongest received signal, which will result in the most precise zone assignment.

The system’s proclaimed emergency location is determined by the zone name of the winning anchor. The gateway’s operation is managed by four firmware constants. The UDP port is fixed at 4210, which is the shared port that the gateway constantly keeps an eye on and where all anchor nodes transmit their reports. The gateway uses the emergency window, which is set to 1,000 ms, to collect and compare anchor reports before determining a zone resolution for a particular emergency. The collection period for recording routine, non-emergency activities is the same as the safe window, which is 1,000 ms. The gateway automatically initiates a new safe state logging window every two seconds, regardless of whether an emergency has occurred, because the safe log period is 2,000 ms. This guarantees samples that are both positive and negative. This guarantees that the output dataset contains both positive and negative samples for balanced model training.

Within its fundamental function, the gateway manages three primary processing activities concurrently. First, there is emergency zone resolution: the gateway initiates a 1,000 ms aggregation window when the first UDP packet with a new sequence number appears. It gathers all anchor reports related to that incident during this period and continuously monitors the report with the strongest smoothed RSSI. After the window closes, the gateway uses the zone and anchor information from the winning report to send a row to a CSV file with an emergency status of 1. The second task is safe state logging: every 2,000 ms, the gateway opens a 1,000 ms window to gather any anchor reports coming from regular BLE advertisements and, when the window expires, writes a CSV row with a status of 0 using the zone of the strongest anchor. The third task is RSSI comparison: every time a new UDP packet arrives during an active window, the gateway checks the new RSSI value against the current best. If the new value is higher, meaning the signal is stronger and the anchor is likely closer to the person, it takes the place of the current best report as the frontrunner for zone assignment.

Within each 1,000 ms aggregation window, multiple anchors might report hearing the same emergency event. The gateway establishes the emergency location through a simple strongest signal approach that operates under three specific circumstances. If only one anchor reports within the aggregation window, that anchor’s zone is automatically chosen as the emergency location. When more than one anchor reports, the gateway selects the anchor with the greatest smoothed RSSI value. This is due to the fact that a less negative RSSI denotes better signal reception and, consequently, a greater physical proximity to the individual. When two anchors report identical smoothed RSSI readings in an unusual situation, the winner is determined by arrival order. This method avoids the computational overhead of trilateration or fingerprint-based methods while maintaining sufficient accuracy for zone-level localization in typical indoor scenarios.

**Figure 5:**
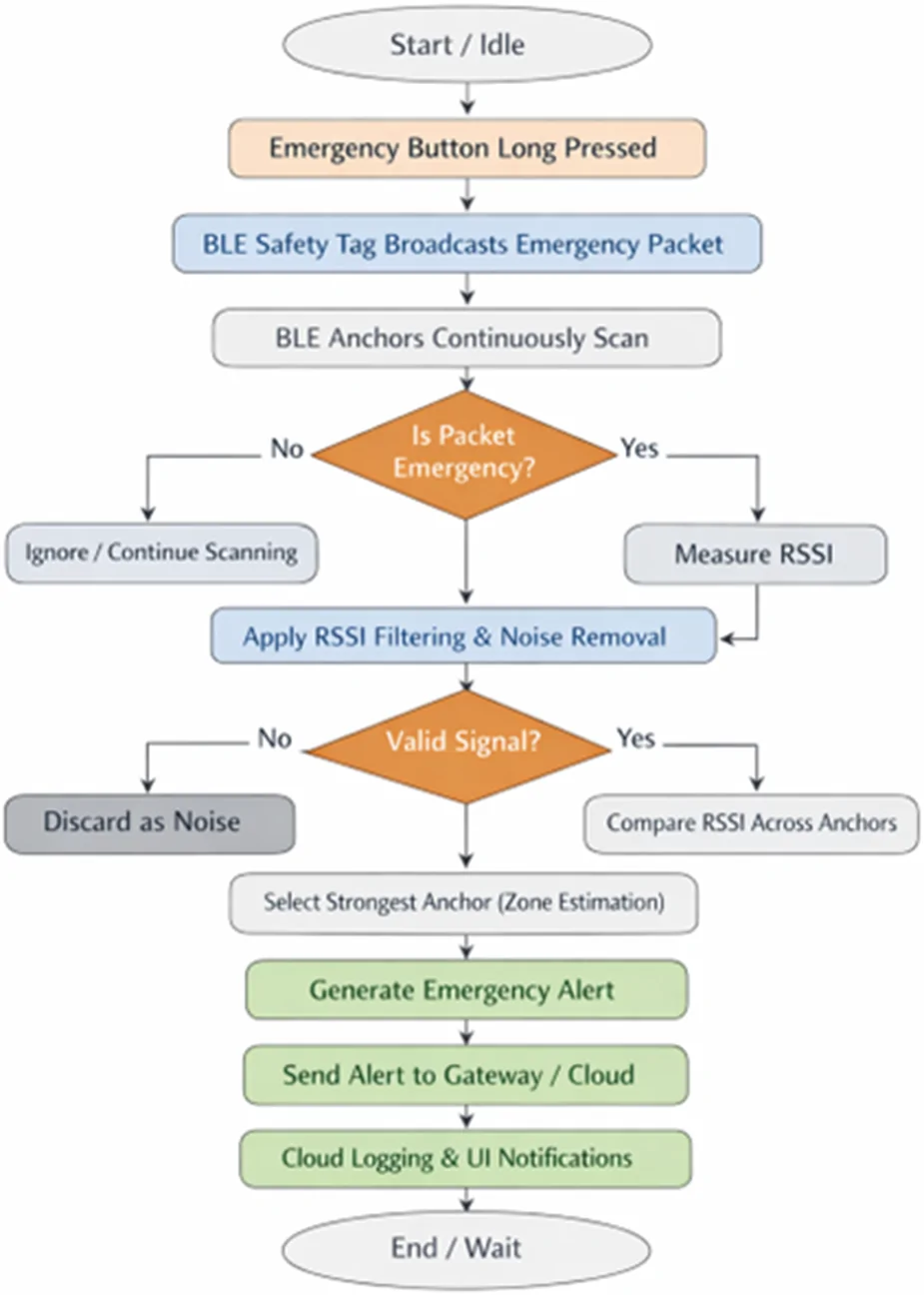
BLE emergency detection flow. From the moment the button is hit to the gateway’s final zone resolution, this figure shows the entire emergency pipeline. BLE packet validation, RSSI filtering, anchor-level deduplication, UDP reporting, and zone identification are all steps in the process. This picture illustrates how the system filters away interference at several stages by clearly showing the steps where packets that are duplicates or not emergencies are discarded.

The detection process, as shown in Figure 5, starts with all nodes resting in an idle state, powered and listening. If someone presses the emergency button and holds it for at least 2,000 milliseconds, the Safety Tag begins sending out iBeacon emergency packets. The BLE Anchor Nodes, which are constantly scanning with a 99 percent duty cycle, pick up these packets and check the safety status flag. Any packets not marked as emergency are immediately discarded. For those that are emergency packets, each anchor measures the raw RSSI, applies sequence deduplication and EMA smoothing (α = 0.25), and then sends a structured UDP report to the gateway. The gateway gathers all reports from the anchors within a 1,000 ms timeframe, identifies the anchor with the strongest smoothed RSSI as the one nearest the person, and pinpoints that anchor’s zone as the emergency location. Once the alert has been sent, the system goes back to being idle, ready for the next happening.

### 3.3 Federated learning pipeline

**Figure 6:**
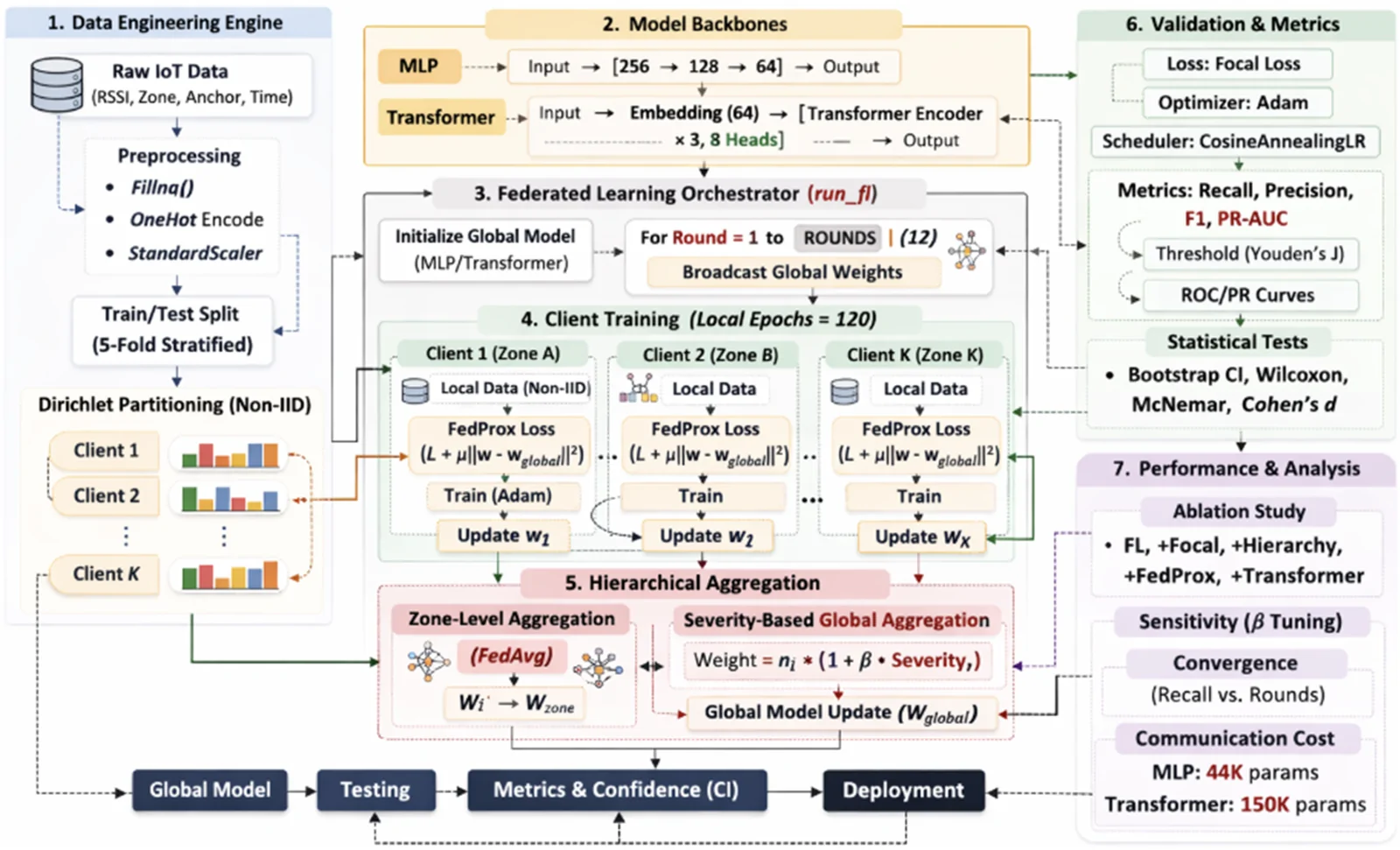
Overall architecture of the FL pipeline. The process lays out the complete federated learning flow using seven key parts: preparing the data, picking the core model setting up the federated coordination, handling local training on each client, combining the results in layers, checking the validity with metrics, and looking closely at how well it works. The chart clearly traces the journey from raw IoT Bluetooth telemetry all the way to a final, combined model that respects privacy and is shared across many distributed anchor clients.

The overall architecture diagram of the federated learning pipeline is described in Figure 6.

#### 3.3.1 Architectures

Two base architectures are used across all five federated configurations:

#### 3.3.2 Multilayer perceptron

**Figure 7:**
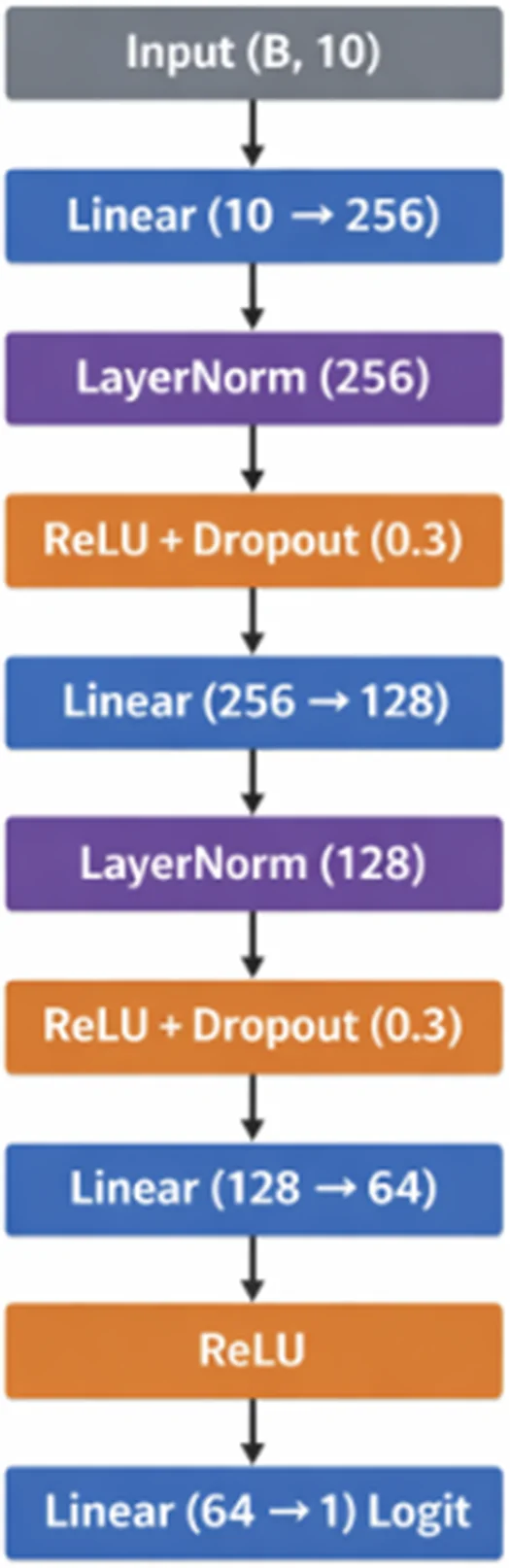
Layer diagram for MLP architecture. This diagram illustrates how the nine-layer Multi-Layer Perceptron served as the foundation for the first four federated arrangements. Dropout and Layer Normalization are employed to help with regularization as the size is gradually decreased from 256 to 128 to 64 units. Layer Normalization was chosen as a better architectural match than Batch Normalization since using non-IID federated partitioning produces wildly inconsistent batch sizes.

The Multi-Layer Perceptron (MLP) acts as the basic foundation for the first four federated setups. As shown in Figure 7 and Table 7, it features a deep, fully connected structure with three hidden layers and uses Layer Normalization in the first two hidden blocks. Choosing Layer Normalization instead of the usual Batch Normalization was a conscious choice because of the way federated training works. When using Dirichlet partitioning with a low concentration parameter of 0.3, a client’s local data can be very uneven, meaning some batches might contain just one sample. Batch Normalization needs at least two samples to calculate running batch statistics and would fail in these situations. Layer Normalization, however, normalizes across the feature dimension for each sample independently. This makes it reliable with any batch size and more suitable for federated systems where each client has a unique, maybe quite extreme, data distribution. The design conducts Layer Normalization, a ReLU activation, and dropout with a 0.3 probability after linearly projecting the 10-dimensional input to 256 units. For a projection down to 128 units, this setup is replicated using Layer Normalization, ReLU, and dropout. A third linear layer projects to 64 units using ReLU without dropout or normalization, while a final linear layer maps to a single output logit. The sigmoid activation is not included into the model itself; rather, it is applied externally during inference to predict probability and internally by the Focal Loss function during training. There are 44,801 trainable parameters in all.

**Table 7:** MLP architecture (input dimension = 10).

Layer	Operation	Output shape	Notes
1	Linear(10 → 256)	(B, 256)	Learnable weights and bias
2	LayerNorm(256)	(B, 256)	Per-sample feature normalization
3	ReLU + Dropout (0.3)	(B, 256)	Activation and stochastic regularization
4	Linear(256 → 128)	(B, 128)	–
5	LayerNorm(128)	(B, 128)	–
6	ReLU + Dropout (0.3)	(B, 128)	–
7	Linear(128 → 64)	(B, 64)	
8	ReLU	(B, 64)	No dropout at this stage
9	Linear(64 → 1)	(B, 1)	Raw logit output

#### 3.3.3 Transformer

**Figure 8:**
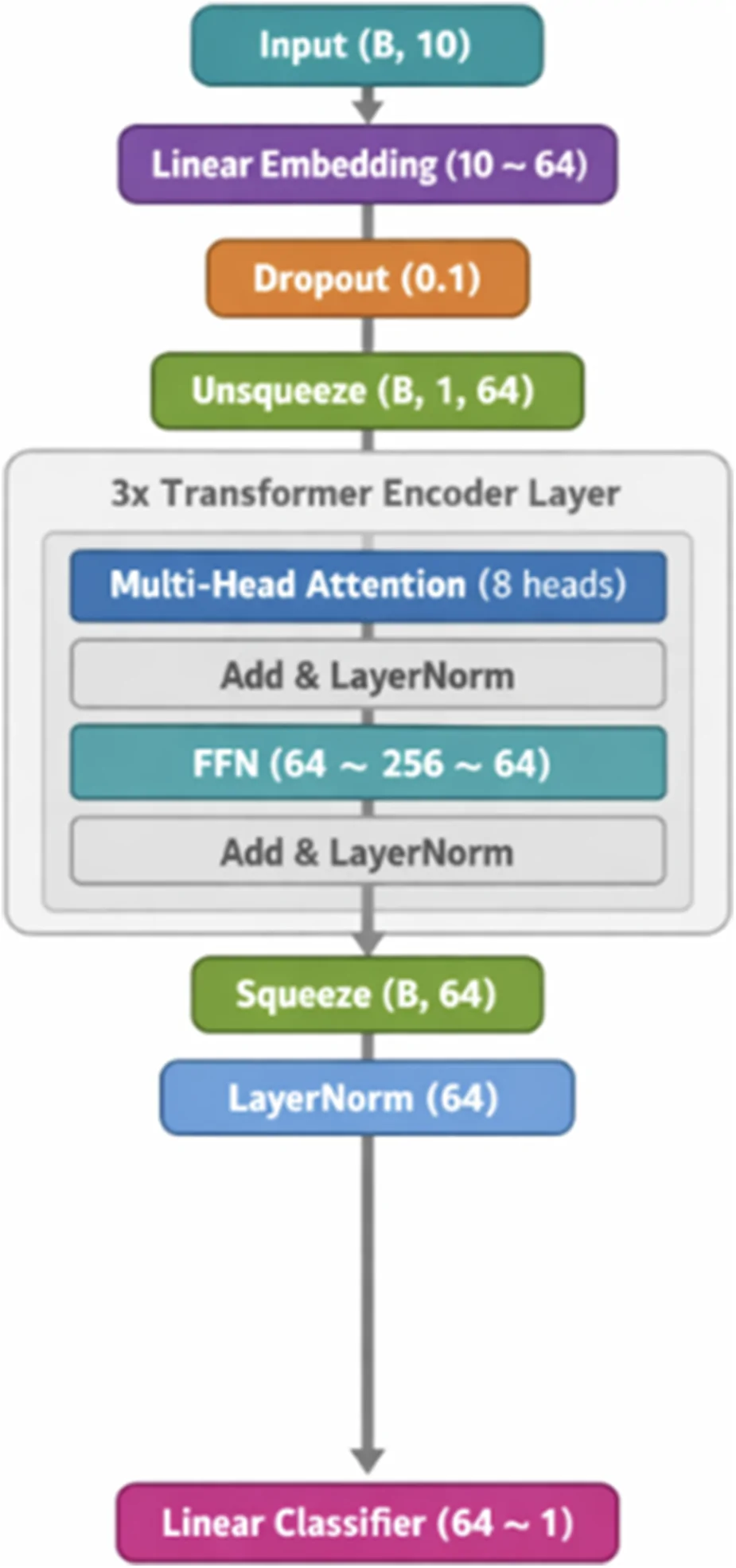
Layer diagram for transformer model. The final SA-HFL setup uses a Transformer structure. This includes a linear embedding layer, positional dropout, three layered Transformer Encoder Layers with 8-head multi-head self-attention, and a simple linear classifier at the end. The self-attention feature of this design lets it pick up on complex relationships between features like RSSI, zonal encoding, and user details, which regular feedforward layers struggle to model well.

The fifth and last configuration being investigated replaces the basic Multi-Layer Perceptron (MLP) as the main engine with the Transformer model, which is the topic of this discussion. In contrast to the MLP, which uses a sequence of simple, independent linear steps to process the feature vector, the Transformer incorporates a self-attention mechanism. This mechanism allows every individual feature to consider every other feature at the same time, thereby capturing complex relationships and dependencies that a simple feedforward design cannot handle. According to Table 8, the model starts with a linear embedding layer that converts the 10-dimensional input into a 64-dimensional representation. A positional dropout of 0.1 is immediately applied after this embedding to help stabilize the representation before it moves into the encoder. The embedded vector is then expanded (unsqueezed) to create a sequence of length 1, which is the necessary format for the standard Transformer encoder. This single-token approach treats the entire feature vector as one unit, and the multi-head self-attention on this single unit essentially performs a learned, multi-subspace projection of the features. This however does not mean a temporal sequence of RSSI is being modeled, the length-1 sequence is simply the format in which the transformer encoder expects. The attention head actually learns how the ten input features, RSSI, zone, anchor and user encodings, weight and inform one another within a single reading rather than how a signal evolves over time. The encoder itself is made up of three layers stacked on top of each other. Each of these layers contains two main parts: a multi-head self-attention mechanism with 8 attention heads and a head size of 8, and a position-wise feed-forward network. This feed-forward network involves a linear expansion from 64 to 256 dimensions, followed by the ReLU activation function, and then a linear reduction back to 64 dimensions. A residual connection and Layer Normalization follow each part, and a dropout of 0.1 is used after both the attention output and the feed-forward output. The batch first convention is used, meaning the batch dimension is the first dimension in all data structures. Once the encoder finishes, the sequence dimension is eliminated by squeezing the tensor, and a final Layer Normalization is applied to the 64-dimensional output before it goes through a single linear classifier that yields one logit value. Just like with the MLP, the sigmoid function is applied outside the model by the loss function. This model has a total of 150,849 trainable parameters. The layers are presented in Figure 8.

**Table 8:** Transformer architecture (input dimension = 10).

Layer	Operation	Output shape	Notes
1	Linear embedding (10 → 64)	(B, 64)	Projects input to model dimension
2	Dropout (0.1)	(B, 64)	Positional dropout
3	Unsqueeze (dim = 1)	(B, 1, 64)	Creates sequence dimension
4–6	3 × TransformerEncoderLayer	(B, 1, 64)	8 heads, FFN dim 256, dropout 0.1
7	Squeeze (dim = 1)	(B, 64)	Removes sequence dimension
8	LayerNorm (64)	(B, 64)	Post-encoder normalization
9	Linear classifier (64 → 1)	(B, 1)	Raw logit output

#### 3.3.4 Loss function–focal loss

Standard binary cross entropy assigns the same importance to all misclassified examples. If a dataset has the normal class making up 60 percent of the total, the easy-to-classify normal examples will drown out the signal in the gradient. Consequently, the prediction system could achieve a deceptively high training accuracy simply by predicting the majority class for every input. This situation is especially dangerous in an emergency detection system. Missing a true emergency, or a false negative, has much more severe consequences than a false alarm. Focal Loss was introduced specifically to solve this problem. It adjusts the standard cross entropy by multiplying it with a modifying factor that dynamically adjusts how much each example contributes to the loss. This adjustment is based on how certain the prediction system is about that specific example. According to Equation 4

$$L_{FL}(p_{t})=\alpha (1-p_{t}{)}^{\gamma }\;\text{BCE}(p_{t})$$ (4)

The prediction system’s predicted probability for the true class, after the sigmoid function, is $p_{t}=\sigma (\text{logit})$. The standard binary cross entropy is $\text{BCE}(p_{t})=-\log\;(p_{t})$. A class balancing weight, α=0.75, gives more emphasis to the less frequent emergency class compared to the normal class. The focusing exponent is γ=2.0. If the prediction system is already sure about a sample, meaning $p_{t}$ is near 1, the modulating factor $(1-p_{t}{)}^{\gamma }$ gets close to zero, essentially minimizing that sample’s contribution to the loss. If the prediction system is unsure or wrong, with $p_{t}$ near 0, the modulating factor gets close to 1, and the full cross entropy loss applies. This approach makes the optimizer concentrate its gradient updates on the tougher, more informative samples. In this system, these are mainly the emergency cases close to the decision boundary. The computation is done using logit level binary cross entropy, which combines the sigmoid and logarithm into a single, more stable operation, and the final loss is averaged across the batch.

#### 3.3.5 Non-IID data partitioning

In a real world anchor setup, different nodes see totally different data distributions. An anchor in a busy hallway runs into far more usual things than one stuck in a rarely used storeroom, and emergency situations might happen more often in specific spots. Performance predictions that appear too good to be true would result from training a federated model with data that was the same for every client. The training data is distributed among the three simulated client devices using a Dirichlet distribution to replicate this variation in a realistic manner. According to Equation 5:

$$p^{\left(c\right)}∼\text{Dirichlet}(\alpha \cdot 1_{K}),\;\alpha =0.3,\;K=3$$ (5)

For each class c (normal or emergency), a percentage vector $p^{\left(c\right)}=[p_{1}^{\left(c\right)},p_{2}^{\left(c\right)},p_{3}^{\left(c\right)}]$ is chosen from a symmetric Dirichlet distribution using a concentration parameter α=0.3. Samples from that class are subsequently distributed to the three clients based on these ratios. The resulting distributions show considerable variety as a result of the low concentration value of 0.3: one client might receive most samples for a given class, while another would receive comparatively few. This is comparable to the uneven distribution of emergency circumstances across all anchor points in the real world. Every class must have a minimum of one sample per client. By doing this, problematic partitions that might disrupt local training—where a client receives no samples of a class—are avoided. Additionally, rather than being set once for the entire fold, the Dirichlet partitioning is redrawn in each federation phase. Similar to the real-world scenario where the distribution of events viewed by each anchor varies over time, this continuous repartitioning introduces round-level data variance that further strains the stability of each federated solution.

#### 3.3.6 Federated aggregation strategies

We examine five distinct federated learning configurations. By introducing one additional component, each configuration expands upon the previous one. All five configurations use the same Focal Loss function, the same data division technique Dirichlet partitioning, and the same training parameters; the only variations are in the underlying network structure and the way the results are aggregated.

#### 3.3.7 FedAvg (baseline)

The standard setup employs the classic Federated Averaging method. In each round of communication, the global model is sent out to all three clients. Each client then trains locally on its data, which has been divided using a Dirichlet partition, for a set number of epochs. After this local training, the client models are returned to the server, which then calculates a new global model by taking a weighted average of all the client parameters. According to Equation 6:

$$w^{\left(t+1\right)}=\sum_{k=1}^{K}\frac{n_{k}}{N}w_{k}^{\left(t\right)}$$ (6)

where $n_{k}$ is the number of examples available for training on client k, $N=\sum_{k}n_{k}$ is the total number of examples across all clients, $w_{k}^{\left(t\right)}$ shows client k’s model settings after local training at round t, and K=3. This weighting makes sure that clients with more data have a proportionally bigger say in the final global model. No extra smoothing is put in place, and combining the results is straightforward, without any group-level hierarchy.

#### 3.3.8 FedProx

FedProx improves upon FedAvg by adding a constraint to each client’s training. This constraint, called a proximal regularization term, is there to keep the client’s model close to the current global model as it trains on its own. When the data isn’t distributed in a similar way across all clients (non-IID), letting clients train without this limit can cause their models to start moving in conflicting directions. This issue is known as client drift, and it makes the process of reaching a stable global model much harder. According to Equation 7:

$$L_{\text{local}}=L_{\text{FL}}(w_{k})+\frac{\mu }{2}{\left\|w_{k}-w^{\left(t\right)}\right\|}^{2}$$ (7)

The global model starts the round as $w^{\left(t\right)}$, and a gentle constraint with μ=0.001 is applied. This constraint, called the proximal penalty, is figured out by adding up the squared differences between each local parameter and its corresponding global parameter. This total is then added to the Focal Loss before backpropagation. A small value for μ gently discourages the local model from wandering off too much without stopping it from learning from its own specific data. The models are combined using the same FedAvg weighted average formula after the local training is complete.

#### 3.3.9 Hierarchical FL (Hier-FL)

The two-step grouping procedure used in hierarchical federated learning adheres to the physical setup’s layout. The three devices are arranged into three distinct sections, one device per area, illustrating how a building’s primary sensors are naturally clustered together. There are two phases to the grouping process:

1. **Zone-level aggregation:** FedAvg is used to generate a model that reflects each zone by combining the various client models.
2. **Global aggregation:** The new global model is obtained by averaging the three models that represent the zones with equal weights of $\frac{1}{3}$ each.

This configuration guarantees that patterns discovered locally, such as in a single room or corridor, are first collected at the zone level before being integrated with patterns from other zones. Combining anchors at the zone level would require averaging multiple clients in a larger experiment with numerous anchors in each zone. Nevertheless, each zone has a single client in the existing three-anchor testbed.

#### 3.3.10 Full SA-HFL (severity-aware hierarchical FL)

At the zone level, the SA-HFL technique modifies the way models are merged. It employs a technique that assigns greater weight according to the severity of the issue rather than just averaging (FedAvg). In a system designed to identify emergencies, the devices (clients) that have actually witnessed more emergency events in a round have better, more relevant data for the task at hand. Because of this, the model updates they create should have a greater influence on the final, combined model. The weight given to each device’s contribution is a combination of two things: the fraction of emergency examples in that device’s local data (severity ratio) and the standard measure based on the total amount of data it holds. According to Equation 8:

$${\tilde{w}}_{k}=\beta \cdot r_{k}+(1-\beta )\cdot \frac{n_{k}}{N}$$ (8)

where the emergency ratio, $r_{k}$, is the count of emergency samples divided by the total samples in client k’s data set. That ratio, along with the regular size-based weight ($n_{k}/N$), determines the client’s overall importance. The severity emphasis parameter, β, which is set to 0.7, manages how much preference is given to emergency cases. With a β of 0.7, a client’s weight is 70 percent determined by its emergency ratio and only 30% by its data volume, which means a strong preference goes to clients with many emergency cases.

As shown in Equation 9, the raw weights are normalized.

$$w_{k}=\frac{{\tilde{w}}_{k}}{\sum_{\;j}{\tilde{w}}_{j}}$$ (9)

The zone-level aggregation is defined in Equation 10.

$$w_{z}^{\left(t+1\right)}=\sum_{k\in z}w_{k}\cdot w_{k}^{\left(t\right)}$$ (10)

#### 3.3.11 Transformer SA-HFL (proposed)

The proposed setup mirrors the Full SA-HFL in how it gathers information: it uses the same two-level hierarchy, the same severity weighted zone level combination with a β of 0.7, the same FedProx regulation with a μ of 0.001, and the same Dirichlet splitting. The only difference is that the MLP core has been replaced with the Transformer architecture described earlier. The Transformer, which has 150,849 parameters, transmits more data in each cycle than the MLP with its 44,801 parameters, but it is better at distinguishing features because of its multi-head self-attention. This attention mechanism learns complex relationships between the starting features that a simple set of straight-line calculations cannot capture. Table 9 shows the overall configurations of the models.

**Table 9:** Progressive configurations of the proposed SA-HFL framework.

Config.	Backbone	Hier.	Severity	FedProx	Params
FedAvg	MLP	No	No	No	44,801
FedProx	MLP	No	No	Yes (μ = 0.001)	44,801
Hier. FL	MLP	Yes (3)	No	No	44,801
Full SA-HFL	MLP	Yes (3)	Yes (β = 0.7)	Yes (μ = 0.001)	44,801
Trans. SA-HFL	Transformer	Yes (3)	Yes (β = 0.7)	Yes (μ = 0.001)	150,849

### 3.4 Training configuration

All five configurations were trained using the same schedule for the federation to ensure a fair comparison. In each of the 12 rounds of communication, the current global model was sent out to all three client devices. Every client then made a new Adam optimizer, starting with a learning rate of $5\times 10^{-4}$, and trained on its local data, which was divided using a Dirichlet partition, for 120 local epochs per round. Throughout these local training cycles, the learning rate followed a Cosine Annealing schedule, which gradually reduced the learning rate from its starting value down to zero, in Equation 11:

$$\eta_{t}=\eta_{\min}+\frac{1}{2}(\eta_{\max}-\eta_{\min})\left(1+\cos\;\left(\frac{t\pi }{T_{\max}}\right)\right)$$ (11)

The learning rate schedule is set by the Equation 12

$$\eta =\eta_{min}+0.5(\eta_{max}-\eta_{min})\left(1+\cos\;\left(\frac{t}{T_{max}}\pi \right)\right)$$ (12)

where $\eta_{\max}=5\times 10^{-4}$, $\eta_{\min}=0$, t is the current local epoch, and $T_{\max}=120$. This specific schedule encourages fast learning at the start of each round and gradually lowers the step size as the client model stabilizes, preventing it from overshooting towards the end of local training. Before every optimization step, gradient clipping was utilized with a maximum norm of 1.0 to prevent the gradient from becoming too large. Because the attention gradients might surge dramatically during the first training rounds, this is particularly crucial for the Transformer model.

To unite the groupings, a five-part stratified cross-validation is employed. This procedure always starts with the same random number, 42. The original sample mix, which was around 60% normal and 40% emergency, was maintained in each of the five portions by splitting the data in this manner. The Dirichlet division is resampled inside each component each time a round takes place. By adding variation at both the round and part levels, this thoroughly examines the efficacy of each group technique. Table 10 describes the hyperparameters of the proposed SA-HFL Framework.

**Table 10:** Training hyperparameters of the proposed SA-HFL framework.

Parameter	Value	Rationale
Communication rounds	12	Sufficient for convergence across configurations
Local epochs per round	120	Enables deep local learning before aggregation
Number of clients	3	Matches 3-anchor physical deployment
Number of zones	3	One zone per anchor in testbed
Optimizer	Adam	Stable adaptive moment estimation
Learning rate	$5\times 10^{-4}$	Balances convergence speed and stability
LR scheduler	Cosine Annealing ($T_{\max}=120$)	Smooth decay over local epochs
Gradient clip norm	1.0	Prevents gradient explosion (Transformer)
FedProx coefficient (μ)	0.001	Gentle drift control
Severity weight (β)	0.7	Emphasizes emergency-rich clients
Dirichlet concentration (α)	0.3	Moderately heterogeneous non-IID splits
Focal loss α	0.75	Up-weights minority emergency class
Focal loss γ	2.0	Suppresses easy-sample gradients
Cross-validation folds	5 (stratified)	Robust performance estimation
Bootstrap resamples	1,000	95% confidence interval estimation
Random seed	42	Ensures full reproducibility

The SA-HFL framework enables numerous devices to train a shared model while maintaining the privacy of their data, as demonstrated by Algorithm 1. It gives more importance to emergency-related data and combines models in a hierarchical way to make the system more reliable.

Algorithm 1 **Severity-Aware Hierarchical Federated Learning (SA-HFL) Algorithm.**

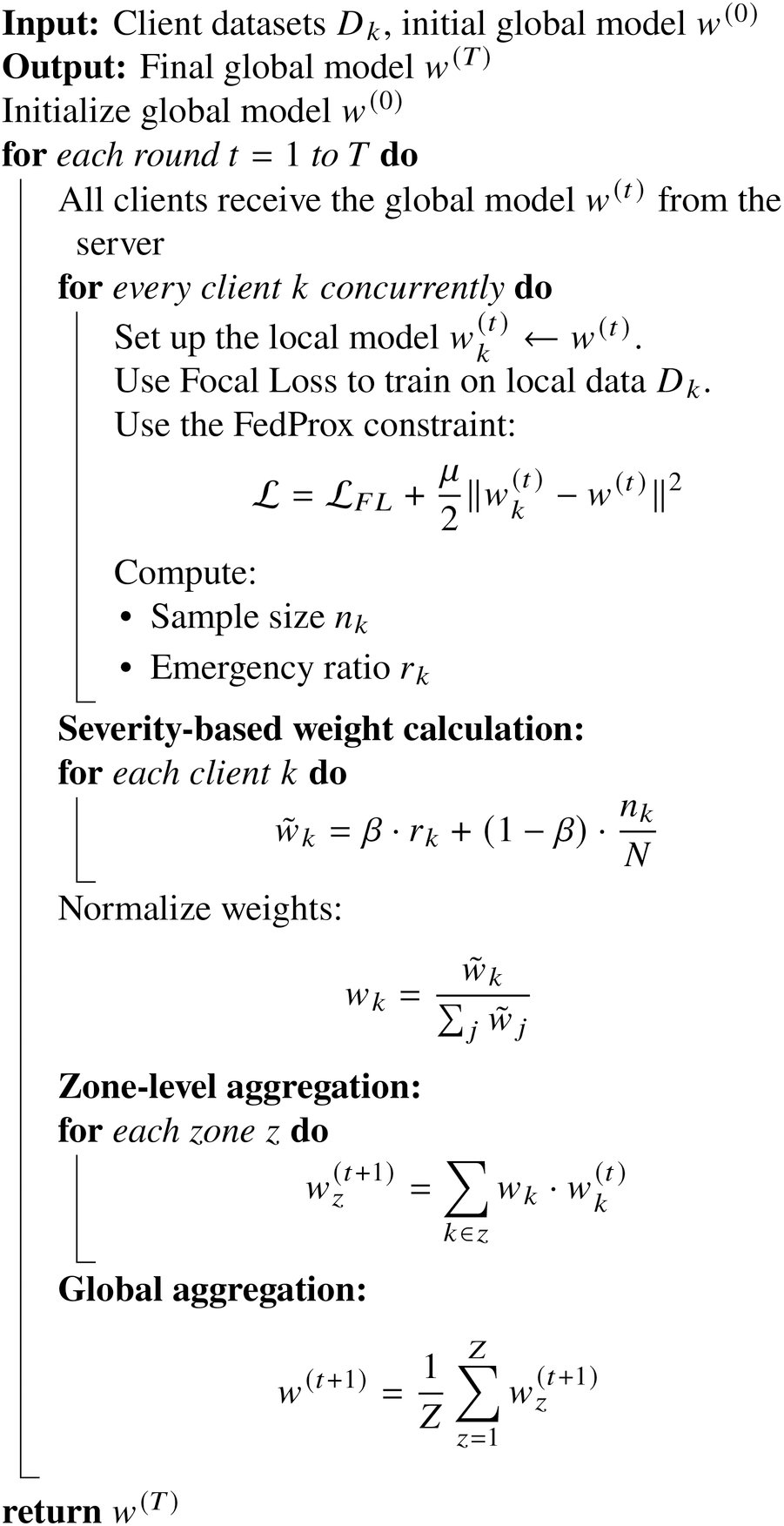

### 3.5 Extended hierarchical configuration and deployment robustness

In order to replicate the actual three-anchor testbed, the primary setup mentioned above makes use of three clients, one for each zone. Also, additional simulated study with nine clients, has been carried out, three per zone, in order to further assess the hierarchical aggregation mechanism at a larger scale. This allows zone-level aggregation to incorporate several independently trained local models prior to global aggregation. This extended configuration is an extra simulated evaluation that is divided using the same Dirichlet technique (α = 0.3) with K = 9; it does not replace the hardware-matched primary setup.

Transformer SA-HFL configuration was evaluated under two additional stress conditions to further assess realism: communication loss, where a successfully trained update is lost in transit to the aggregator with probability $p_{\mathrm{drop}}\in \{0.10,\;0.20,\;0.30\}$, which represents network unreliability different from device failure, and client dropout, where a scheduled client fails to train or return an update with probability $p_{\mathrm{loss}}\in \{0.10,\;0.20,\;0.30\}$. Any zone with zero surviving updates in a particular round is skipped in zone-level and global aggregate.

### 3.6 Privacy scope and limitations

Patient IDs, location data, and raw BLE telemetry never leave the original device; MIBES only exchanges model parameters between clients and the aggregator. Although it does not provide a formal privacy guarantee, its data-local design lowers the attack surface in comparison to centralized data collection. No formal mitigations like differential privacy or safe aggregation since model parameter changes was used, especially gradients from models trained on tiny local datasets, can reveal information about the underlying data through membership inference or gradient inversion attacks. In order to give measurable privacy guarantees, MIBES was characterized as data-local rather than privacy-preserving in the formal sense and point to the inclusion of safe aggregation techniques or differential privacy as crucial future work.

## 4 Results and discussion

### 4.1 Task scope and metric reporting

Because zone resolution is deterministic rather than learned, standard classification metrics reported in Table 11 apply only to the emergency-recognition task. Zone-resolution reliability is instead characterized separately in the physical deployment evaluation (Table 16), which reflects the conditions under which the strongest-RSSI rule operates, and, where applicable, a direct localization accuracy against known ground truth.

**Table 11:** Comprehensive main results comparison (5-fold CV mean).

Model	Acc	Rec	Prec	F1	AUC	Bal.Acc	MCC
FedAvg (baseline)	0.9930	0.9868	0.9971	0.9919	0.9997	0.9923	0.9858
FedProx	0.9938	0.9908	0.9948	0.9928	0.9997	0.9934	0.9873
Hierarchical-FL	0.9922	0.9874	0.9949	0.9911	0.9996	0.9917	0.9843
SA-HFL (severity-aware)	0.9940	0.9914	0.9949	0.9931	0.9997	0.9937	0.9878
**Transformer SA-HFL**	**0.9952**	**0.9908**	**0.9983**	**0.9945**	**0.9997**	**0.9947**	**0.9904**

Bold values indicate the best-performing result in each evaluation metric.

### 4.2 Software simulation: transformer-based SA-HFL performance

To be able to simulate a real-world distributed clinical scenario, the Transformer-based SA-HFL model underwent extensive testing under challenging statistical scenarios during the evaluation phase. A carefully selected dataset of 4,000 patient samples with a high emergency ratio of 43.5% (1,741 emergency cases compared to 2,259 routine samples) was used for the simulation. This ratio was intentionally selected to represent a busy medical facility with frequent but localized critical alerts.

#### 4.2.1 Experimental environment and dependencies

Every experiment used an NVIDIA Tesla T4 GPU and a GPU-accelerated kernel running Python 3.12.12. To ensure that every partitioning, initialization, and fold-splitting decision could be fully replicated, the random seeds were fixed everywhere (np.random.seed(42), torch.manual_seed(42)).

#### 4.2.2 Dataset composition and feature engineering

A multi-stage feature engineering pipeline was used to prepare the raw BLE telemetry for the Transformer backbone. A 10-dimensional feature space was created by expanding the initial vector, which combined:

- **Temporal Metadata:** To monitor the course of events, Unix Timestamps were included.
- **Spatial Context:** One-Hot Encoding was used to transform the zonal identifiers and anchor node indices. The distinct radio-frequency (RF) characteristics unique to each room were captured in this step.
- **Signal Intelligence:** Temporal smoothing was performed using sequence numbers and Raw Received Signal Strength Indicator (RSSI) values.

To normalize all numerical features, a StandardScaler was used. By ensuring that the Transformer’s self-attention layers could effectively compute similarities, this phase prevented the influence of different feature scales.

#### 4.2.3 High-fidelity comparative performance

SA-HFL was compared to four alternative approaches in order to show the strength of the proposed framework in terms of both clinical results and technical performance. These include the standard FedAvg, a severity-aware MLP-based SA-HFL, FedProx (which deals with differences in data distribution), and Hierarchical FL (which groups data by zone). Table 11 shows the average scores obtained from a five-fold stratified cross-validation.

The proposed SA-HFL approach achieved the best results with the highest precision (0.9983) and MCC (0.9904). This shows how effectively the attention part removes radio-frequency noise, which frequently causes simpler models like FedAvg to become unstable. In medical contexts, the Matthew’s Correlation Coefficient (MCC) is the main metric used to assess safety. Compared to just using accuracy, it provides a more fair assessment of how well something is classified when the dataset is uneven. Compared to standard MLP setups, the Transformer structure’s MCC of 0.9904 is a major improvement. This improved performance comes from the Transformer’s self-attention ability, that learns weighted dependencies among the 10 dimensional input features (RSSI, zonal encoding, anchor encoding and user information) at a given instant, this helps in capturing feature interactions that a fixed feed forward layer applies uniformly and independently. This is a feature relation benefit rather than a temporal one,as the current input representation does not include a multi step RSSI time series.

**Figure 9:**
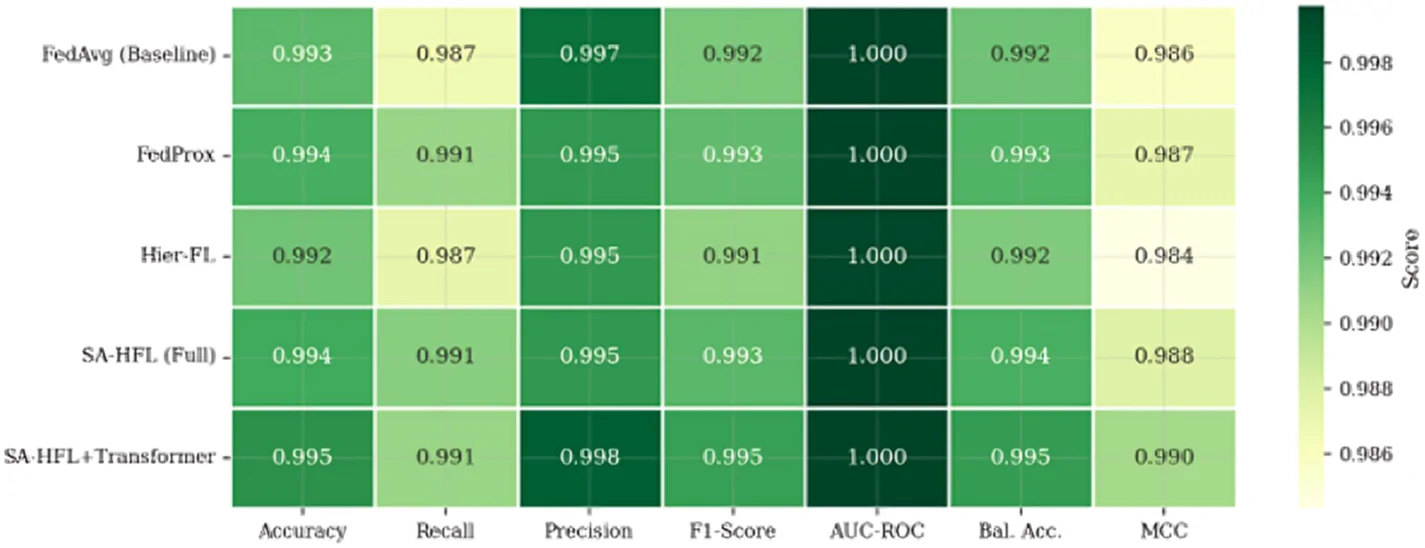
Heatmap of Performance Metrics showing relative strengths across models. Accuracy, Recall, Precision, F1 Score, AUC-ROC, Balanced Accuracy, and MCC are the seven metrics used in the matrix to compare all five configurations. Better performance is indicated by darker green. In the Precision and MCC columns, the Transformer SA-HFL row stands out with extremely dark coloring, demonstrating that it outperforms the standard FedAvg in safety-critical classification.

Figure 9 shows a visual a representation of Table 11. The X-axis shows the evaluation matrix and the Y-axis shows the model setups. The color blend (going from red/white to dark green) illustrates how concentrated the performance is. In tasks requiring highly dependable identification, the SA-HFL row performs better than the standard FedAvg baseline, as shown by its strong coloring in the Precision and MCC columns.

#### 4.2.4 Statistical significance and reliability analysis

Even small advances in safety-critical IoT need statistical support. The 95% Confidence Intervals were calculated using a bootstrap technique (*N* = 1000). Cohen’s d was also computed to determine the impact of severity weighting and hierarchy.

Table 12 shows the mathematical reliability of the results under repeated resampling. The Wilcoxon signed rank test nor the paired t-test reached statistical significance at the 0.05 level for any configuration (SA-HFL: Wilcoxon *p* = 0.2500, *t*-test *p* = 0.2078; Transformer: Wilcoxon *p* = 0.2500, *t*-test *p* = 0.2709), which is expected as the number of paired folds (*n* = 5) available for these tests were limited. This limited statistical power shows that the observed recall improvements over FedAvg cannot be considered as statistically significant differences. However the Cohen’s d metric, which measures Effect Size is not dependent on sample size in the same way and is above 0.74 for both the severity aware MLP and the Transformer versions, this indicates a moderate to large practical effect. Combined together, these results show a consistent and practically meaningful trend toward improved recall, but confirmation with larger number of independent trials or folds would be required to establish statistical significance. Fold level McNemar tests (*p* = 1.00 for several folds, p>0.12 overall) further show that the classifier does not show structural instability or overfitting and maintains a consistent classification pattern across data splits.

**Table 12:** Advanced statistical justification and reliability trial.

Model	Rec ± Std	95% CI (Boot)	Wilcoxon p	*t*-test p	Cohen’s d
FedAvg	0.9868 ± 0.0053	[0.9834, 0.9920]	–	–	–
FedProx	0.9908 ± 0.0022	[0.9891, 0.9925]	0.1875	0.1991	0.885
Hier-FL	0.9874 ± 0.0080	[0.9805, 0.9943]	0.8750	0.9077	0.076
SA-HFL	0.9914 ± 0.0041	[0.9879, 0.9948]	0.2500	0.2078	0.867
**Transformer**	**0.9908 ± 0.0042**	**[0.9868, 0.9937]**	**0.2500**	**0.2709**	**0.748**

Bold values indicate the best-performing result in each statistical metric. 95% confidence intervals were estimated using bootstrap resampling (*N* = 1000).

**Figure 10:**
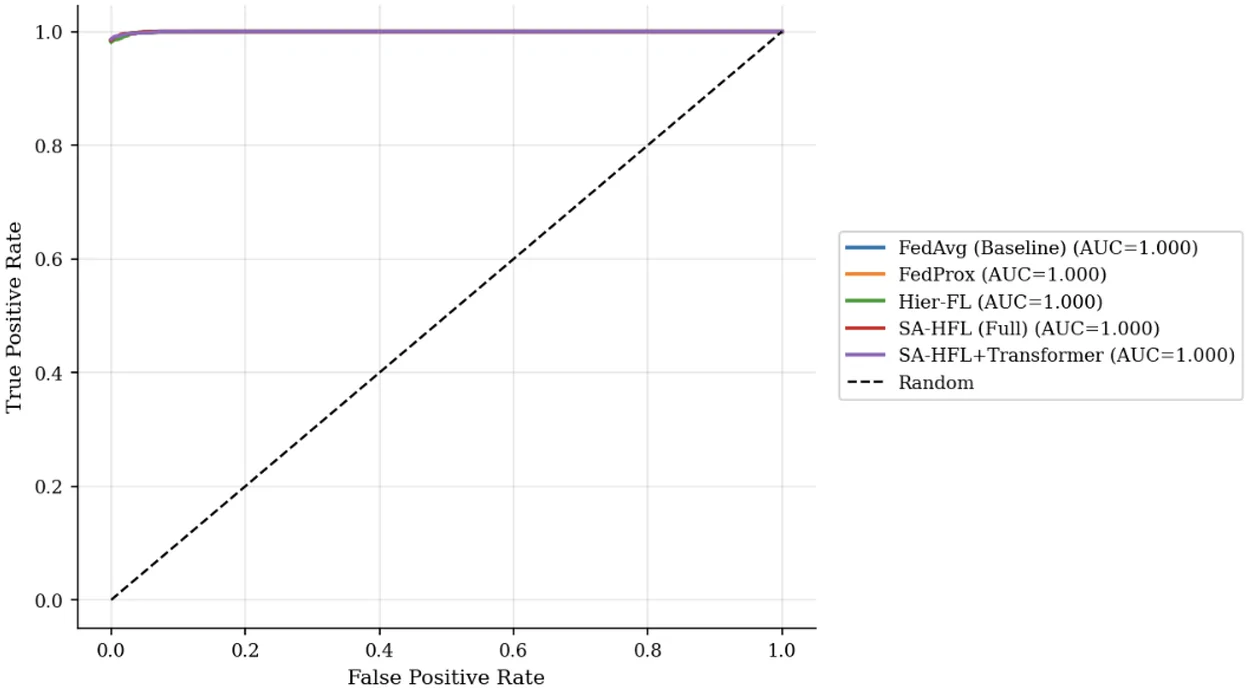
AUC-ROC overlays across the 5 configurations. The Receiver Operating Characteristic curves for each of the five models shows that all configurations cluster close to the upper-left corner at an AUC of approximately 1.000. This shows excellent class separation in all federated strategies. Even though it is very difficult to differentiate between the curves at this scale, when a complementing precision-recall analysis is used, slight differences become significant in a clinical setting.

**Figure 11:**
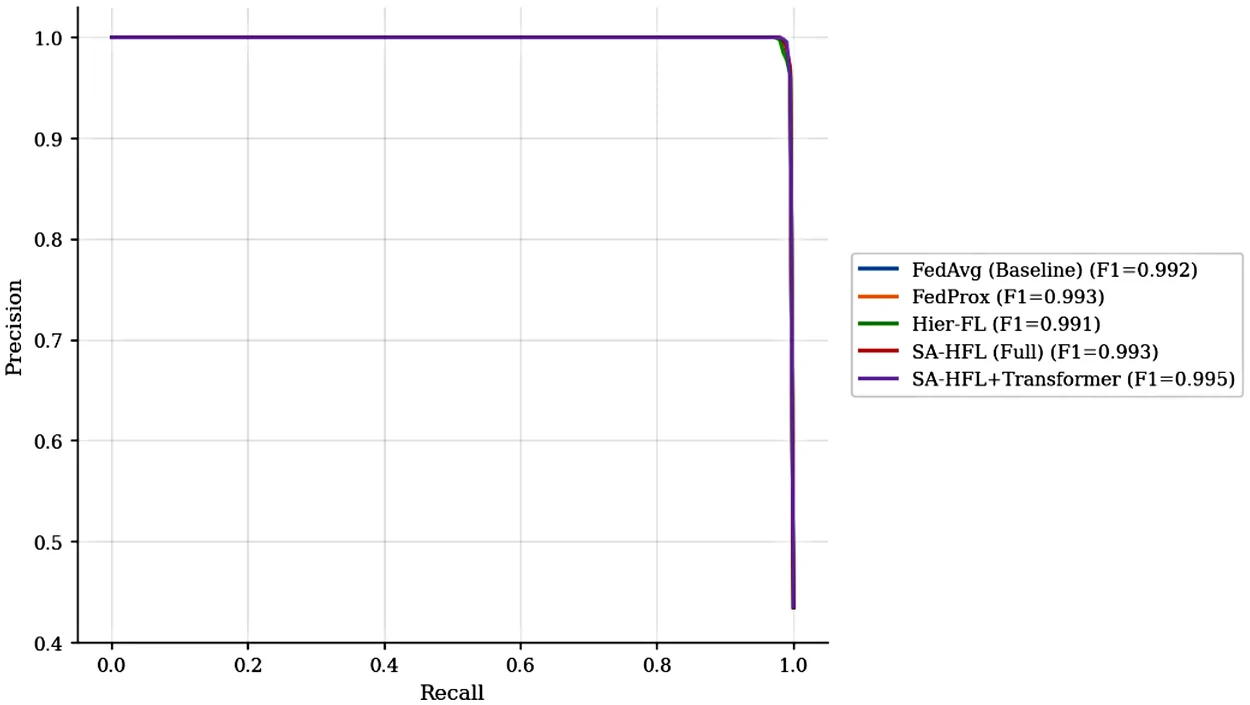
Precision-Recall overlays across the 5 configurations. This plot, shows Precision against Recall curves for each of the five settings, this provides a clearer picture of how the models perform on the challenging emergency dataset. The best is the Transformer SA-HFL curve (orange), which maintains the highest precision even at high recall. By reducing the amount of false emergency alerts, this directly addresses the health issue of alert fatigue.

Figure 10 shows the True Positive Rate plotted against the False Positive Rate, the almost perfect overlap of the curves in the upper-left corner (AUC = 0.999) shows how well each model can differentiate between classes. Plotting the precision vs. recall plot in Figure 11 gives a better understanding of the imbalanced medical dataset. Compared to the other curves, the Transformer’s orange curve stays closest to the top-right corner, showing that it maintains almost perfect precision even at high recall. Preventing constant “Alert Fatigue” that occurs in hospitals that is necessary.

#### 4.2.5 Technical ablation: dissecting the value of SA-HFL

An ablation study was used to evaluate each system component’s contribution. The most significant gain in Recall was obtained by moving from standard Binary Cross Entropy (BCE) to Focal Loss (A2), which effectively made the local models prioritize those “harder” emergency situations, as shown in Table 13.

**Table 13:** Component-wise ablation performance.

Configuration step	Mean recall	Mean F1-score	Mean AUC
A1: Baseline (FL + BCE)	0.9897	0.9939	0.9997
A2: +Focal Loss (γ=2)	0.9925	0.9945	0.9998
A3: +Hierarchical Zonal Agg	0.9914	0.9931	0.9997
A4: +Severity Weighting (β)	0.9874	0.9908	0.9994
A5: +FedProx (Local Reg)	0.9931	0.9928	0.9997
**A6: +Transformer Backbone**	**0.9902**	**0.9931**	**0.9997**

Bold values indicate the best-performing result in each evaluation metric.

The analysis included determining how much the system responded to the Severity Emphasis parameter (β). The whole model was found to be optimal for medical emergencies when β was kept between 0.6 and 0.95, without completely disturbing the “No Emergency” baseline group.

#### 4.2.6 Sensitivity to severity weighting (β)

**Figure 12:**
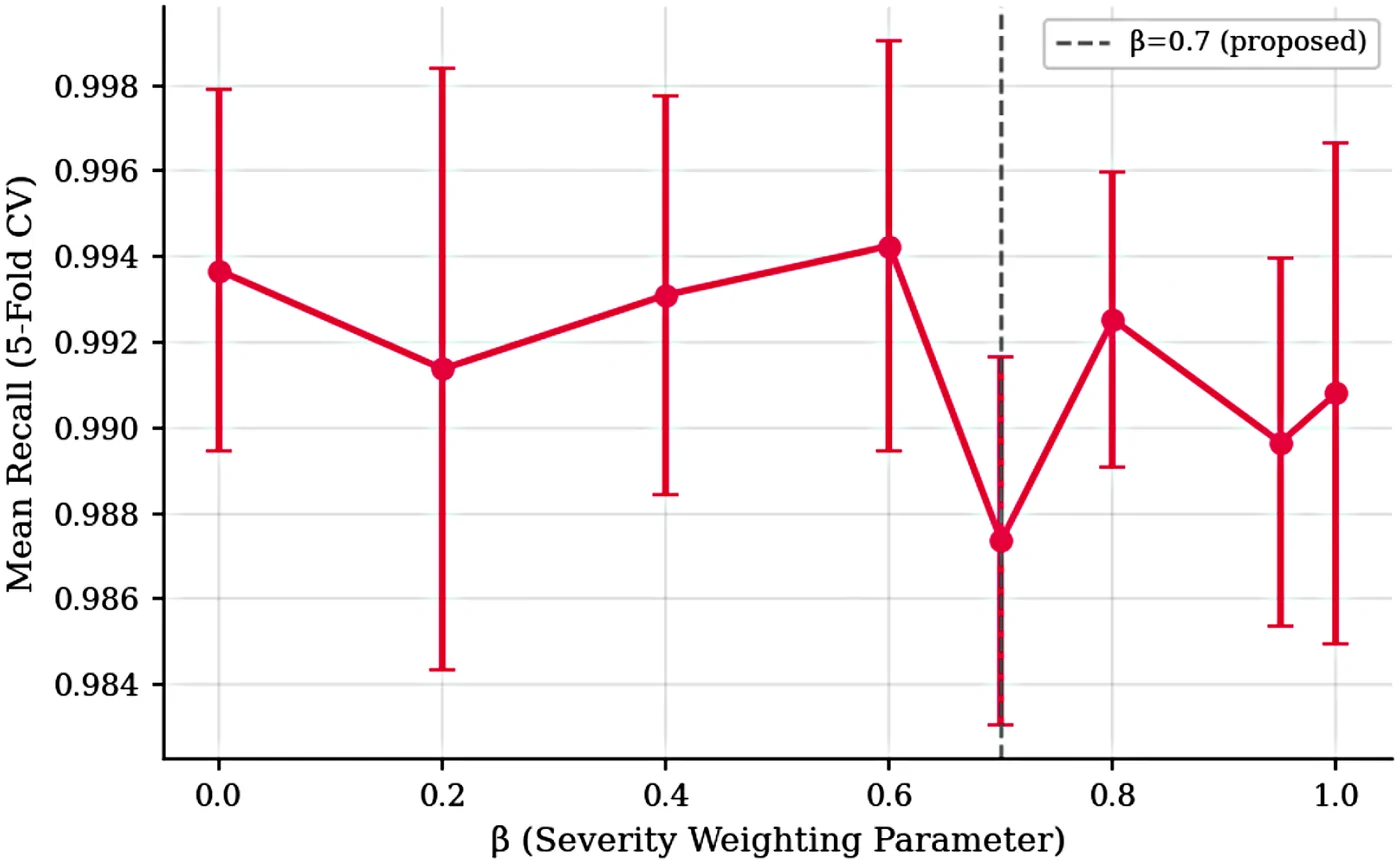
Beta sensitivity analysis. The impact of the severity-aware weighting parameter β on the overall recall of the model was examined over its whole range [0.0, 1.0]. It was found that the recall was at its highest point at β = 0.60 (0.9948) and then it gradually decreased towards the ends of the range. In order to ensure that the aggregation strongly benefits clients with several emergencies, a final operational value of β = 0.7 was selected. This value aligns with the system’s mandate to emphasize safety.

Based on Figure 12, the impact of the severity-aware parameter β on global model sensitivity was examined over the range [0.0,1.0].

Although β=0.60 gave the best recall (0.9948), the operating standard was retained as β=0.7 for all reported experiments. This choice was made to give critical alerts from clients experiencing serious issues the highest priority, guaranteeing that the system as a whole moves toward a secure state.

#### 4.2.7 Confusion matrix and error distribution

**Figure 13:**
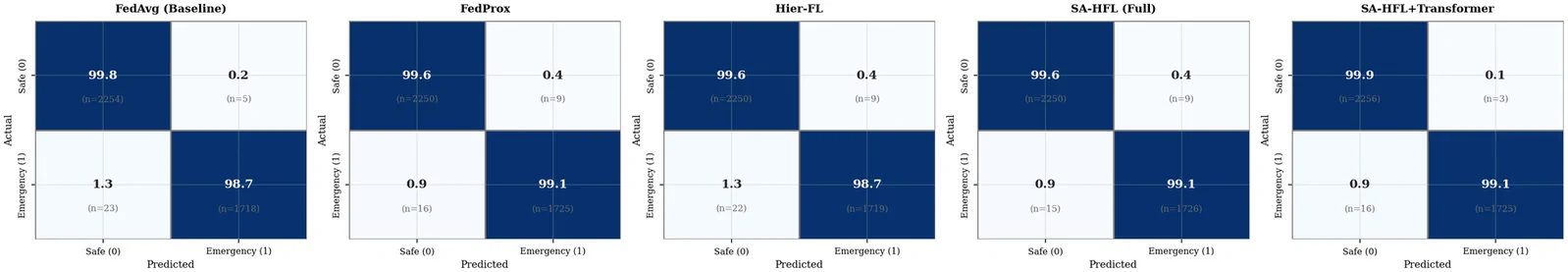
Confusion matrices for the evaluated models: FedAvg, FedProx, Hier-FL, SA-HFL, and SA-HFL-Transformer. The comparison shows normalized confusion matrices for all five setups for FedAvg, FedProx, Hier-FL, SA-HFL, and Transformer SA-HFL by directly comparing how often they produce false positives and false negatives. The Transformer SA-HFL matrix stands out with the fewest false positives (it misclassifies about 0.1% of normal samples), proving that it is the most effective in reducing unnecessary emergency alerts in clinical settings.

The confusion matrices for each of the five federated models shown in Figure 13 were carefully analyzed to determine the configurations’ reliability in a clinical scenario. The results clearly showed that the Transformer SA-HFL produced the fewest false alarms (False Positives), which is crucial for preventing alarm fatigue in demanding medical settings.

All of the models achieve high accuracy but when compared, the Transformer-based model excels at rejecting noise, and removing it by 99.9% . This shows that out of 2,259 samples, it only flags an error three times.

#### 4.2.8 Leakage-controlled feature ablation

To verify that model performance derives from genuine BLE signal intelligence rather than artifacts of the data-generation pipeline, a leakage-controlled ablation was conducted in which the timestamp, user, and sequence seq fields were removed, retaining only RSSI, zone, and anchor as model inputs. This ablation was motivated by an observed structural property of the dataset: the seq field is populated only for emergency-labeled samples and is missing for all normal samples, making it a near-perfect proxy for the label rather than genuine signal information. Table 14 reports the results of this ablation using a centralized MLP classifier trained under the same Focal Loss objective, evaluated via a single stratified train/test split.

**Table 14:** Leakage-controlled feature ablation.

Feature set	Acc	Rec	MCC
Full 10-feature (original)	0.9830	0.9609	0.9659
seq + zone + anchor (no RSSI)	0.9725	0.9368	0.9452
RSSI + zone + anchor (no seq/timestamp/user)	0.5617	0.0149	−0.0047

This ablation reveals that seq alone, without any RSSI information, reproduces nearly the full reported performance (MCC 0.9452 vs. 0.9659), while RSSI in isolation performs at chance level (MCC ≈ 0). This indicates that the strong performance reported in Table 11 derives substantially from the seq field’s structural correlation with the label, a byproduct of the gateway logging pipeline rather than genuine RSSI-based signal intelligence. The finding must be reported transparently rather than obscuring it, and identify closing this gap, through richer temporal RSSI features, multi-anchor signal fusion, or logging seq consistently for both classes, as a critical requirement for future work before the reported metrics can be interpreted as evidence of genuine BLE-based emergency recognition.

#### 4.2.9 Generalization validation: spatial and temporal holdout

Beyond random 5-fold cross-validation, it was evaluated with generalization under two stricter holdout protocols: leave-one-zone-out, training on two zones and testing on the entirely unseen third zone, and a chronological holdout, training on the earliest 70% of samples by timestamp and testing on the most recent 30%. Both use the full feature set and a centralized MLP classifier under the same training protocol as the leakage ablation above.

According to Table 15, MCC remains between 0.876 and 0.926 when the model is evaluated on a zone entirely absent from training, indicating that performance does not depend on having seen a specific zone’s characteristics during training. Temporal generalization, however, is substantially weaker. Under the chronological holdout, accuracy falls to 0.5658, precision to 0.5123, and MCC to 0.3071, despite recall remaining high (0.9890). This gap suggests that random 5-fold cross-validation, which shuffles samples across the full time range, may mask a real distribution shift over time that is not captured by the original evaluation protocol, and that this shift may partly compound the seq-related leakage identified above. This limitation was reported directly rather than relying solely on the random-split results in Table 11, and identify temporally-aware validation as necessary for future evaluations of this system.

**Table 15:** Leave-one-zone-out spatial generalization.

Held-out zone	Acc	Rec	Prec	MCC
Zone_A	0.9565	0.9754	0.9264	0.9126
Zone_B	0.9342	1.0000	0.8661	0.8757
Zone_C	0.9631	0.9391	0.9788	0.9259

### 4.3 MIBES analysis

Although the software model achieved nearly perfect metrics, MIBES has significant physical layer challenges in the real world. As a result, during the course of five minutes, an ESP32 Safety Tag was used to collect raw networking metrics.

#### 4.3.1 Quantifying the networking-signal gap

The actual experiment differed greatly from the simulations. Table 16 shows that 54.29% of the system’s packets were lost.

**Table 16:** Physical deployment performance summary.

Networking metric	Observational value
Experimental duration	300.00 s
Total diagnostic packets	35 Packets
Successful reception	16 Packets
Estimated packet loss	54.29%
RSSI stability (mean)	−75.66 dBm
Network throughput	0.12 pkt/s
Inter-arrival delay (Jitter)	8256.06 ms (Avg)
Maximum observed latency	31333.00 ms

The log captures the performance of the ESP32 safety tag. The 54.29% packet loss and 8,256 ms jitter are significant values because they show the challenging signal conditions at a medical facility where significant telemetry gaps are caused by multipath fading and hardware limitations. The SA-HFL model must handle the signal floor, which is set by the average RSSI of −75.66 dBm.

Given the high packet loss and significant jitter averaging 8.2 s, it made perfect sense to go with an EMA-based RSSI smoothing layer (α=0.25) and a 1,000 ms Temporal Aggregation window. Without those layers, random RSSI spikes would continuously trigger the system’s decision-making rather than real clinical events. The average RSSI of −75.66 dBm suggests the ESP32 is a fair distance from the anchor nodes, which is why having that noise-resilient filtering is so essential.

### 4.4 Architectural scalability and efficiency

The communication overhead, which is the main sticking point for federated healthcare systems, was evaluated last. Although the Transformer-based backbone is more complex, it only raised the communication cost per round to 1.81 MB (up from 0.54 MB for MLP).

Because the Transformer model is so much more precise and statistically dependable, a total bandwidth need of 21.72 MB, as shown in Table 17, is a small price to pay for the huge safety advantages.

**Table 17:** Parametric and communication efficiency analysis.

Efficiency metric	MLP SA-HFL	Transformer SA-HFL
Total model parameters	44,801	150,849
Local data exchange (round)	0.54 MB	1.81 MB
Total bandwidth (12 rounds)	6.45 MB	21.72 MB
**Relative recall gain**	**+0.46%**	**+0.40%**

Bold values indicate the best-performing result in each evaluation metric.

**Figure 14:**
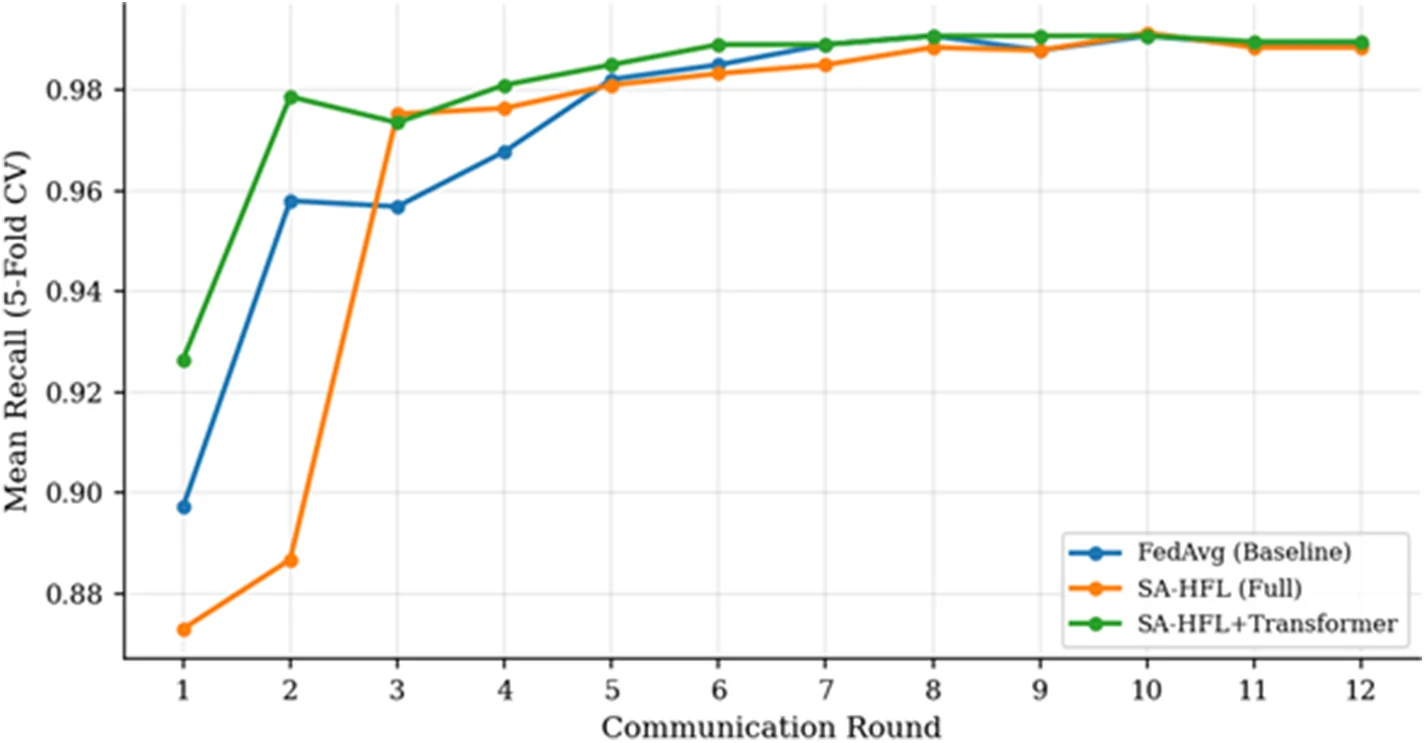
Convergence dynamics plot. The graph shows the mean recall for the FedAvg baseline, SA-HFL (Full), and Transformer SA-HFL setups over 12 federated communication rounds. In the early rounds, the Transformer model shows the fastest improvement and settles down first. This suggests that, while having more complex parameters than the more simpler MLP-based options, it learns efficiently and converges fast.

**Figure 15:**
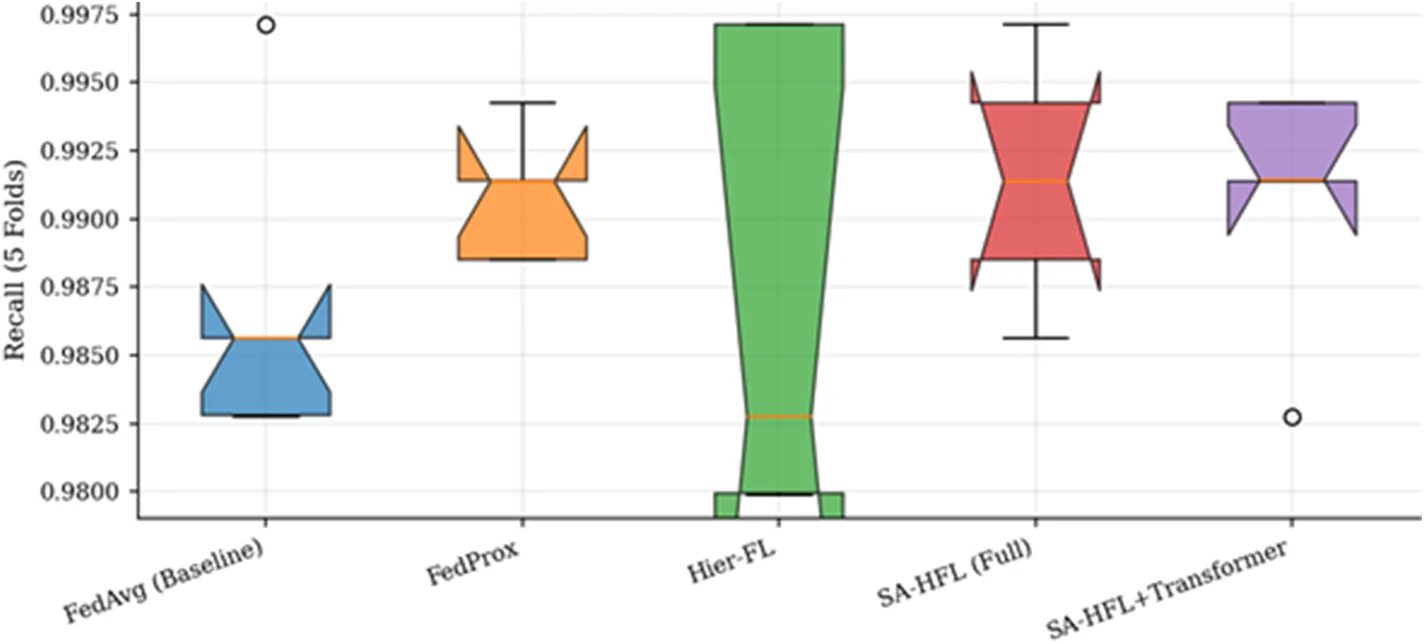
Box plot for cross-validation stability analysis. Box plots are used to show the variation in recall scores across five stratified cross-validation folds for all federated settings, providing a clear overview of the average performance and how much it has changed from one fold to the next. With the smallest variation between the first and third quartiles and the fewest extreme values, the Transformer SA-HFL model truly showed its reliable and consistent results regardless of how the data was divided into different regions.

Recall is tracked over 12 federated rounds as shown in Figure 14. The Transformer model (Orange) is seen to rise sharply and level out quickly, showing high training effectiveness. The distribution of recall for each of the five cross-validation folds is compared in Figure 15. Strong reliability is shown by the Transformer model’s concentrated distribution and thin whiskers, which show that the system operates consistently regardless of how the institutional data is divided.

In conclusion, a system that combines edge-side EMA filtering with Transformer-based severity-aware federated learning is both highly sensitive (99.08% recall) and robust enough to handle the inherent instability of Bluetooth Low Energy in complex medical settings.

### 4.5 Robustness under realistic federated conditions

To address concerns about the scale of the hierarchical setup, a supplementary nine-client (three-per-zone) simulated study alongside the primary three-client results in Table 11. Recall in this extended setting was comparable across all five configurations (FedAvg 0.9879, FedProx 0.9885, Hier-FL 0.9885, SA-HFL 0.9874, Transformer SA-HFL 0.9891), confirming the larger simulated hierarchy remains stable. We also assessed the Transformer SA-HFL setup under client dropout and communication loss in the same nine-client scenario (Table 18). At dropout probabilities of 0.10, 0.20, and 0.30, recall changed from 0.9891 (no stress) to 0.9891, 0.9885, and 0.9805, while MCC changed from 0.9873 to 0.9883, 0.9868, and 0.9787. Recall was 0.9879, 0.9897, and 0.9879 under communication loss of 0.10, 0.20, and 0.30, whereas MCC was 0.9883, 0.9873, and 0.9869. Under realistic deployment stress, degradation stayed within about one percentage point in both scenarios, suggesting elegant rather than catastrophic degradation.

**Table 18:** Robustness under client dropout and communication loss.

Condition	Recall	MCC
	(Mean ± Std)	
Ideal	0.9891±0.0042	0.9873
Dropout ($p_{\mathrm{drop}}=0.10$)	0.9891±0.0046	0.9883
Dropout ($p_{\mathrm{drop}}=0.20$)	0.9885±0.0060	0.9868
Dropout ($p_{\mathrm{drop}}=0.30$)	0.9805±0.0042	0.9787
Comm. Loss ($p_{\mathrm{loss}}=0.10$)	0.9879±0.0042	0.9883
Comm. Loss ($p_{\mathrm{loss}}=0.20$)	0.9897±0.0047	0.9873
Comm. Loss ($p_{\mathrm{loss}}=0.30$)	0.9879±0.0091	0.9869

### 4.6 MIBES setup overview

**Figure 16:**
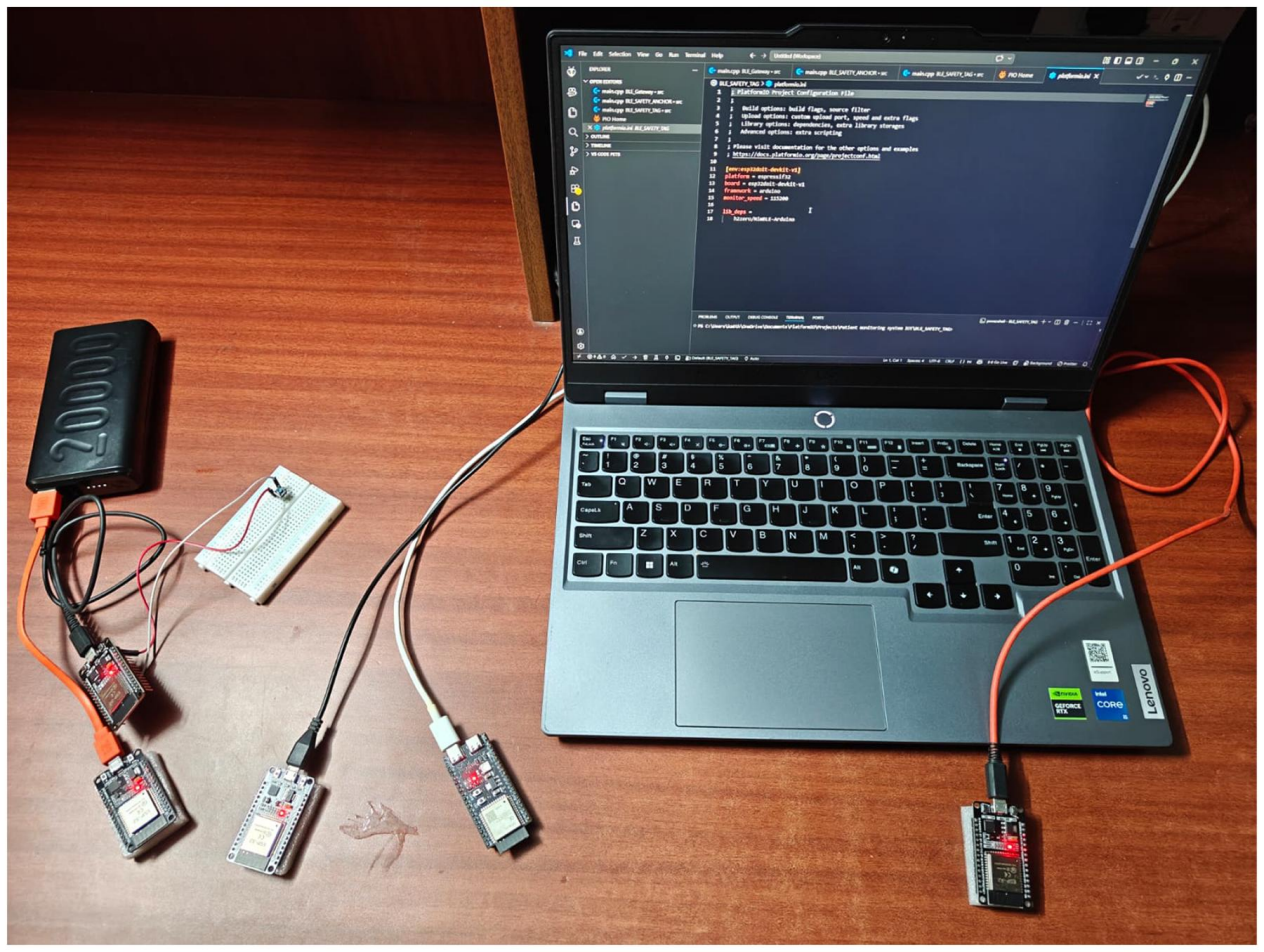
Hardware setup of the MIBES system. The physical testbed implementation of the proposed system is shown here, which includes the central ESP32-S3 Gateway Node receiving UDP telemetry, the fixed BLE Anchor Node in passive scanning mode, and the wearable ESP32 DevKit V1 Safety Tag with its push-button. This real-hardware validation shows that the proposed framework is not just a computer model but a working, deployable system under real indoor radio frequency conditions.

Figure 16 shows the BLE Safety Tag, that is used by individuals. It has an ESP32 connected to a basic push-button on a breadboard. This button is what triggers the emergency. BLE Anchor Node located at predetermined indoor locations, serves as a fixed infrastructure component. This ESP32-based device remains in passive scanning mode, listening for BLE advertisement packets that nearby safety tags may send. The preliminary tasks like packet validation and duplication filtering are handled by the anchor. The system’s main decision-making brain is the Gateway Node. It uses a more powerful ESP32-S3 board and gets UDP packets from the various anchor nodes across the local network.

## 5 Conclusion

MIBES, a reliable three-tier IoT system for privacy-preserving emergency identification in indoor medical settings, was introduced in this research. The proposed solution in simulation, numerically outperformed the standard federated learning baselines, achieving 99.52% accuracy and a MCC of 0.9904, with Focal Loss integration and severity aware weighting (β=0.7) providing a strong sensitivity to emergency events under non-IID conditions, while keeping raw sensor data local to each device through parameter-only sharing, though without a formal privacy guarantee. These headline metrics must be interpreted alongside the leakage-controlled ablation reported in Section 4, which found that a substantial portion of this performance derives from a structural artifact in the dataset’s sequence field rather than genuine RSSI-based signal intelligence, and alongside the generalization validation showing near-zero performance once this artifact is removed. These results should be looked at as an evidence of feasibility rather than clinical readiness. The physical deployment testing of the solution was limited in scope, it included a single 300 s session, 35 diagnostic packets, one wearable safety tag, one user and three fixed anchors and this test showed a packet loss of 54.29%, an average inter-arrival jitter of 8.26 s and a mean RSSI of −75.66 dBm. The EMA smoothing and a 1,000 ms gateway aggregation window were designed to kae the downstream classification pipeline more robust to noises, but a single short session with one user is not sufficient to showcase the reliability of the solution under multi user, multi floor and higher occupancy conditions that is normally present in clinics and hospital environment. MIBES should be regarded as a promising proof of concept architecture than a system that is ready for clinical deployment. To confirm the real world viability of the system extended trails with multiple simultaneous users and safety tags, tested across various indoor layouts and inference conditions and evaluation with clinical staffs to assess the usability and emergency response workflows under realistic clinical conditions is required.

## 6 Future work

Future research aims to improve the MIBES framework’s context-awareness by integrating multi-sensor fusion, particularly by combining biometric telemetry and IMU-based fall detection with BLE signal strength. In order to provide formal privacy guarantees against membership inference attacks, further research may examine the implementation of Differentially Private (DP) methods within the SA-HFL pipeline. Additionally, by using Reinforcement Learning (RL) to dynamically tune scanning duty cycles and EMA parameters in response to changing room occupancy and ambient interference, the architecture’s expansion into intricate multi-floor environments might be examined. Lastly, to confirm the system’s long-term operational dependability and power efficiency in high-density healthcare infrastructures, extensive clinical trials must be carried out. Future physical deployment trials will log ground-truth position across many concurrently active anchors, independent of the RSSI-based rule, to directly evaluate zone-resolution accuracy in addition to the emergency-classification metrics given in this paper. These future trials should also specifically test conditions not covered in the current single-session deployment, including body shadowing from multiple simultaneous occupants, crowded ward environments with elevated ambient RF interference, multi-floor layouts requiring inter-floor anchor coordination, and concurrent emergency triggers from multiple safety tags, with confidence intervals for packet loss and all main classification metrics computed across repeated independent sessions rather than single runs. Independent external validation on separate hardware and at a different physical site remains necessary to establish generalizability beyond the single testbed used in this study.

## Data Availability

The original contributions presented in the study are included in the article/Supplementary Material, further inquiries can be directed to the corresponding author.
